# Characterization of epithelial-mesenchymal transition intermediate/hybrid phenotypes associated to resistance to EGFR inhibitors in non-small cell lung cancer cell lines

**DOI:** 10.18632/oncotarget.21132

**Published:** 2017-09-22

**Authors:** Valentina Fustaino, Dario Presutti, Teresa Colombo, Beatrice Cardinali, Giuliana Papoff, Rossella Brandi, Paola Bertolazzi, Giovanni Felici, Giovina Ruberti

**Affiliations:** ^1^ Institute of Cell Biology and Neurobiology, National Research Council (IBCN-CNR), Monterotondo, Rome, Italy; ^2^ Institute for Systems Analysis and Computer Science “Antonio Ruberti” National Research Council, (IASI-CNR), Rome, Italy; ^3^ Genomics facility of the European Brain Research Institute, “Rita Levi-Montalcini” (EBRI), Rome, Italy; ^4^ SYSBIO Center for Systems Biology, Milan, Italy

**Keywords:** EMT intermediate/hybrid phenotype, NSCLC, EGFR, erlotinib, microarray data

## Abstract

Increasing evidence points to a key role played by epithelial-mesenchymal transition (EMT) in cancer progression and drug resistance. In this study, we used *wet* and *in silico* approaches to investigate whether EMT phenotypes are associated to resistance to target therapy in a non-small cell lung cancer model system harboring activating mutations of the epidermal growth factor receptor. The combination of different analysis techniques allowed us to describe intermediate/hybrid and complete EMT phenotypes respectively in HCC827- and HCC4006-derived drug-resistant human cancer cell lines. Interestingly, intermediate/hybrid EMT phenotypes, a collective cell migration and increased stem-like ability associate to resistance to the epidermal growth factor receptor inhibitor, erlotinib, in HCC827 derived cell lines. Moreover, the use of three complementary approaches for gene expression analysis supported the identification of a small EMT-related gene list, which may have otherwise been overlooked by standard stand-alone methods for gene expression analysis.

## INTRODUCTION

Epithelial-mesenchymal transition (EMT) - wherein epithelial cells depolarize, lose their cell–cell contacts and gain an elongated fibroblast-like or amoeboid shape - plays important roles in embryonic development, tissue repair and cancer biology [1–3]. Several studies, mostly *in vitro*, have supported the hypothesis that during cancer progression tumor cells undergo dynamic and reversible transitions from epithelial to mesenchymal, and from mesenchymal to epithelial (MET) phenotypes and that EMT and MET are required for cancer invasion and metastasis [1, 4]. Moreover, through EMT cancer cells may also acquire stem cell properties and self-renewal capability [5, 6]. The EMT/MET hypothesis as a driving force for cancer invasion and metastasis has been recently challenged by very elegant *in vivo* murine studies [7, 8]. In these studies, EMT was not rate limiting for invasion and metastasis, but rather associated to chemotherapy resistance [7, 8].

Multiple signaling pathways and complex genetic and epigenetic mechanisms regulate the EMT program in normal and neoplastic epithelial tissues [1, 9–12]. Importantly, the EMT is not a binary process and cancer cells with intermediate or hybrid epithelial/mesenchymal (E/M) phenotypes characterized by a mixture of epithelial and mesenchymal traits have been described [13–16]. Intermediate E/M phenotypes may contribute to cancer collective cell migration and cell clusters formation by preservation of cell-cell interactions including epithelial as well as E/M cells. Circulating tumor cell (CTC) clusters have been increasingly observed in the bloodstream of many patients with aggressive malignancies including lung cancer and these clusters have been associated with worse clinical outcomes as compared to the presence of single CTCs [17–19].

Lung cancer is the most frequent cause of cancer-related mortality worldwide leading to over a million deaths each year [20]. Based on histological characteristics, the two principal types of human lung cancer are small cell lung cancer (SCLC) and non-small cell lung cancer (NSCLC). The latter contributes to nearly 85% of lung cancer cases. Identification of all driver oncogene alterations in lung adenocarcinoma and consequently adoption of coherent molecular target therapies are challenging because of a large burden of passenger events per tumor genome [21–23]. However NSCLC patients, whose tumors harbor sensitizing and driving mutations in the epidermal growth factor receptor (EGFR), get a meaningful clinical benefit from EGFR tyrosine kinase inhibitor (TKI) treatments. Unfortunately acquired resistance invariably develops [24, 25]. Importantly, acquired NSCLC resistance has also been associated to EMT [26–29].

In order to investigate the mechanisms of resistance to TKI, we have recently reported the establishment and characterization of NSCLC cell lines resistant to the EGFR inhibitor erlotinib [30].

The effect of TKI target therapy on the selection of intermediate E/M phenotypes in cancer cells is still poorly investigated. Therefore, in this study, we used *wet* and *in silico* approaches to investigate whether E/M phenotypes are associated to erlotinib-resistance in our cellular model system. The combination of different analysis techniques allowed us to describe intermediate and complete EMT phenotypes in HCC827- and HCC4006-derived erlotinib-resistant cell lines respectively. Interestingly, EMT intermediate phenotypes, collective cell migration and increased stem-like ability associate to resistance to target therapy in the erlotinib-resistant HCC827-derived cell lines. Moreover, the use of three complementary approaches for gene expression analysis supported the identification of a small EMT-related gene list, which may have otherwise been overlooked by standard stand-alone methods for gene expression analysis.

## RESULTS

### EMT features analysis of erlotinib-resistant NSCLC cells

Recently, in order to investigate mechanisms leading to resistance to EGFR-targeted therapy, two NSCLC cell lines (HCC827 and HCC4006) have been used to derive *in vitro* models of acquired resistance to the EGFR TKI erlotinib [30]. Both parental cell lines harbor EGFR activating mutations in the tyrosine kinase domain, precisely in exon 19. In particular, the HCC827 cell line carries a deletion in exon 19 (ΔE746-A750) and the HCC4006 carries a deletion (ΔL747-E749) and a point mutation (A750P) in exon 19. Both HCC827 and HCC4006 cell lines are highly sensitive to TKIs targeting the EGFR, while their derived cell lines (i.e: RA1, RA2, RB1, RB1.1, RB2 derived from HCC827 and the RC2.2 derived from HCC4006) are stably resistant to erlotinib (IC_50_ > 10 μM) [30]. Characterization of these erlotinib-resistant cell lines, all negative for the common T790M EGFR mutation, has been previously described [30] and is schematically summarized in Supplementary Table 1.

**Figure 1:**
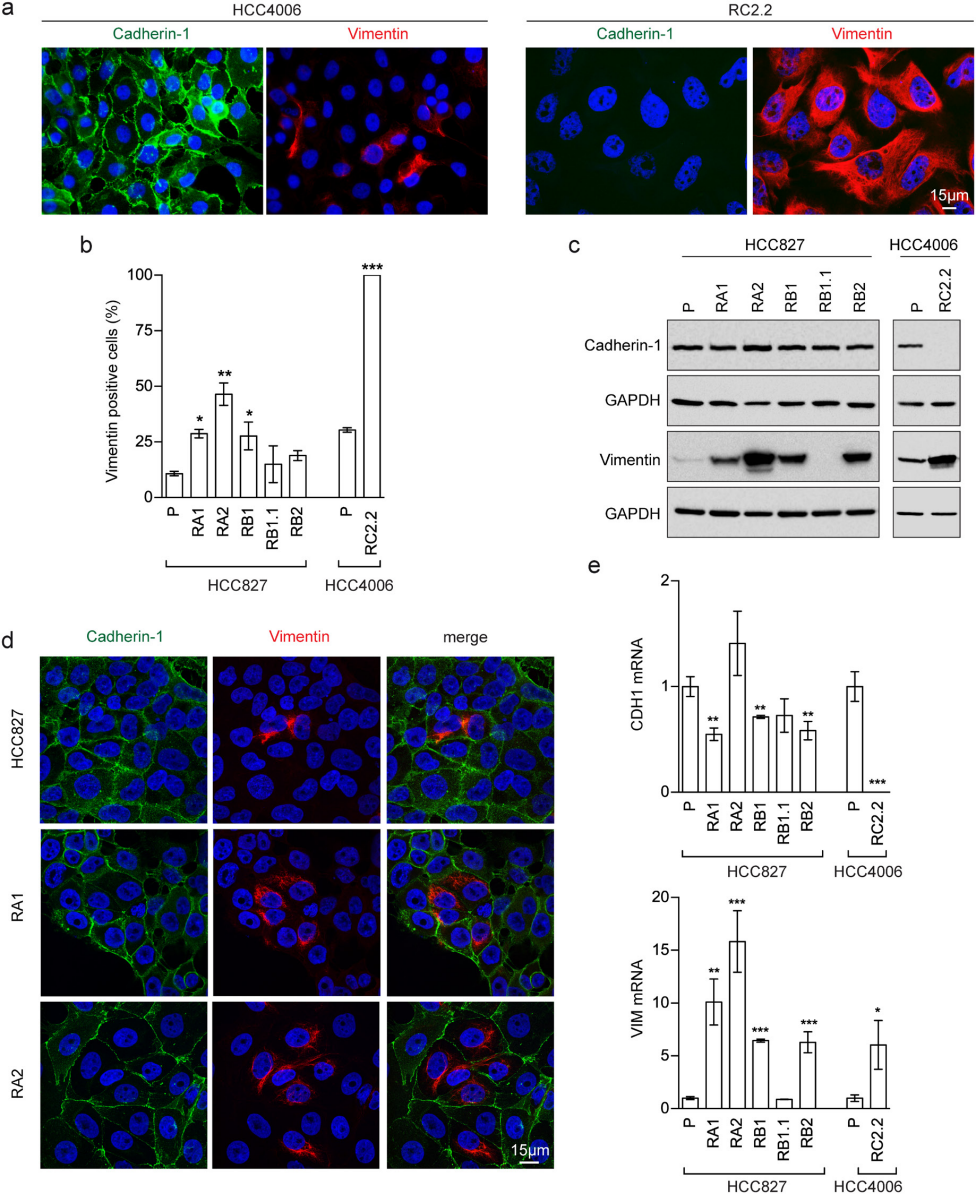
EMT features in erlotinib-resistant NSCLC cell lines **(a)** Fluorescence microscopy analysis of HCC4006 and RC2.2 cell lines stained with specific reactive antibodies for Cadherin-1 (green), Vimentin (red) and Hoechst 33258 for nuclei (blue). **(b)** Histograms represent the percentage of Vimentin positive cells (mean ± SD), obtained by the analysis of at least 10 images with approximately 350-500 nuclei/image for each cell line in at least three independent experiments; **(c)** Western blot analysis of Cadherin-1 and Vimentin in the indicated parental (P) and erlotinib-resistant cell lines; **(d)** Confocal microscopy analysis of cell lines stained with specific reactive antibodies for Cadherin-1 (green) and Vimentin (red) and Hoechst 33258 for nuclei (blue). Images represent the projection of 4-5 selected focal planes spanning 1.6-2 μm of the specimens. Scale bars are indicated; **(e)** qPCR analysis of CDH1 and VIM mRNAsnormalised to rp-L31 mRNA and expressed relative to the levels in parental (P) cell lines set as 1 (mean ± SD). qPCR data are representative of those obtained from three independent analysis. Asterisks in the figure indicate significant t-test p-values relative to the comparison of given derived cell lines with the corresponding parental one. * p < 0.05; ** p < 0.01; *** p < 0.001. Only significant p-values are shown.

**Figure 2:**
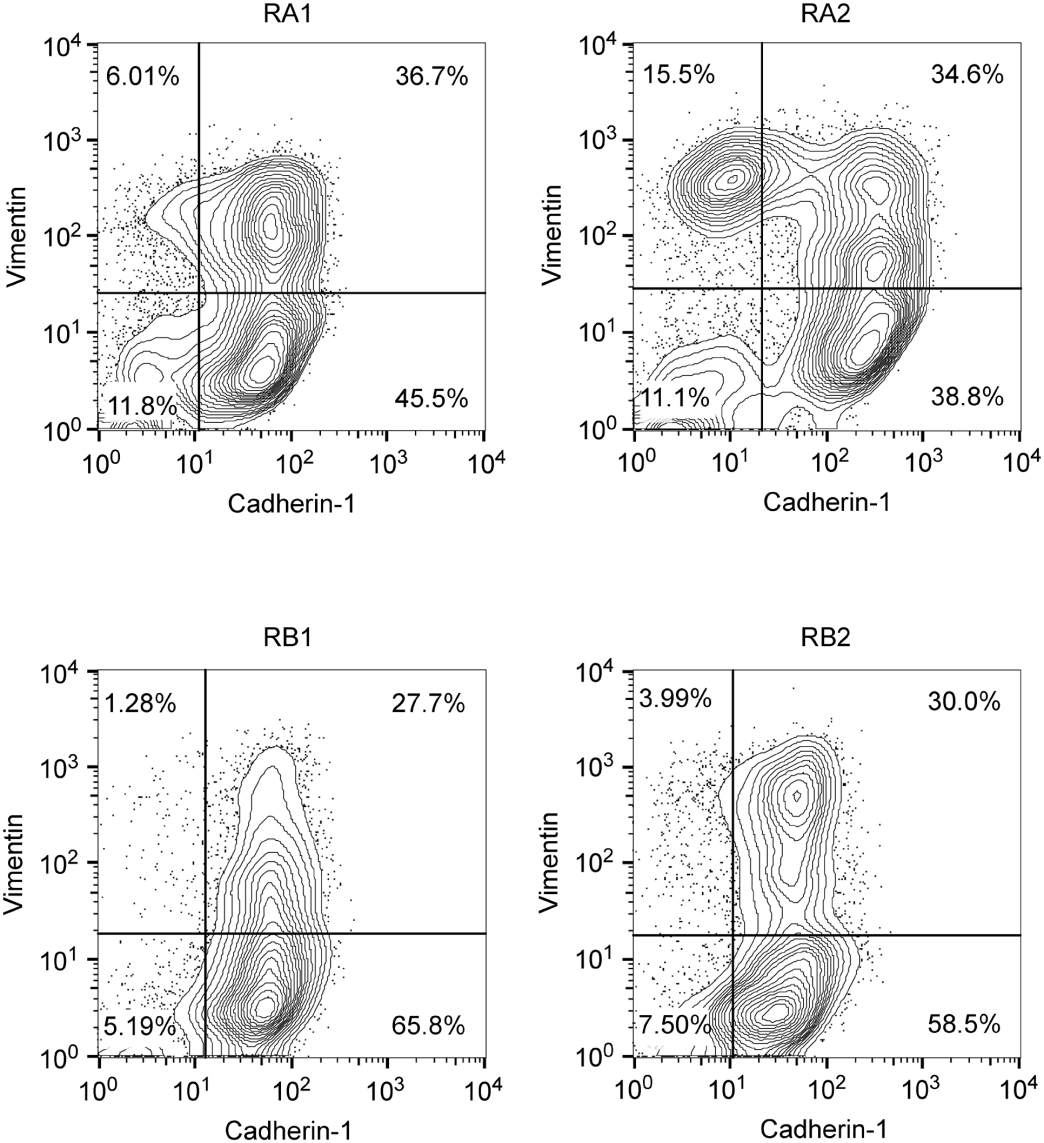
Double-color flow cytometry analysis of erlotinib-resistant NSCLC cell lines Shown are representative contour plots of RA1, RA2, RB1 and RB2 erlotinib-resistant cell line samples stained with Cadherin-1 as epithelial marker (x-axis) and Vimentin as mesenchymal marker (y-axis). Staining with isotype matched antibodies (data not shown) were used to set the gates and calculate the percentage of single positive and double positive subpopulations.

Interestingly, morphological analysis of the erlotinib-resistant NSCLC cells showed the presence of cells with a fibroblast-like cell shape reminiscent of EMT, especially in the RA1, RB1, RB2 and RC2.2 cell lines (Supplementary Figure 1). Indeed, EMT features in the erlotinib-resistant cell lines were detected by assaying the epithelial marker Cadherin-1 (also known as E-cadherin) and the mesenchymal marker Vimentin by different methodologies, such as immunofluorescence and confocal microscopy (Figure 1a, 1b and 1d), western blot (Figure 1c) and mRNA expression analysis (Figure 1e). In particular, RC2.2 cells are all negative for Cadherin-1 and positive for Vimentin, similarly to the HCC4006ER cell line previously described [31] (Figure 1). An increase in the number of Vimentin positive cells compared to parental cell lines is observed not only in the RC2.2 cells but also in RA1, RA2, RB1 and RB2 erlotinib-resistant cell lines at both protein (Figure 1b and 1c) and mRNA levels (Figure 1e), without any notable variation in the Cadherin-1 expression levels. Remarkably, some cells double positive for Cadherin-1 and Vimentin are present in the HCC827 erlotinib-resistant cell lines in particular in RA1 and RA2 (Figure 1d). To quantify numbers of cells with epithelial, mesenchymal or intermediate E/M markers in the cell lines with an higher numbers of Vimentin positive cells, we employed flow cytometry analysis. Indeed subpopulations of intermediate E/M cells positive for both Cadherin-1 and Vimentin markers are present in the HCC827 erlotinib-resistant cell lines RA1 (36.7%), RA2 (34.6%), RB1 (27.7%) and RB2 (30%) (Figure 2).

**Figure 3:**
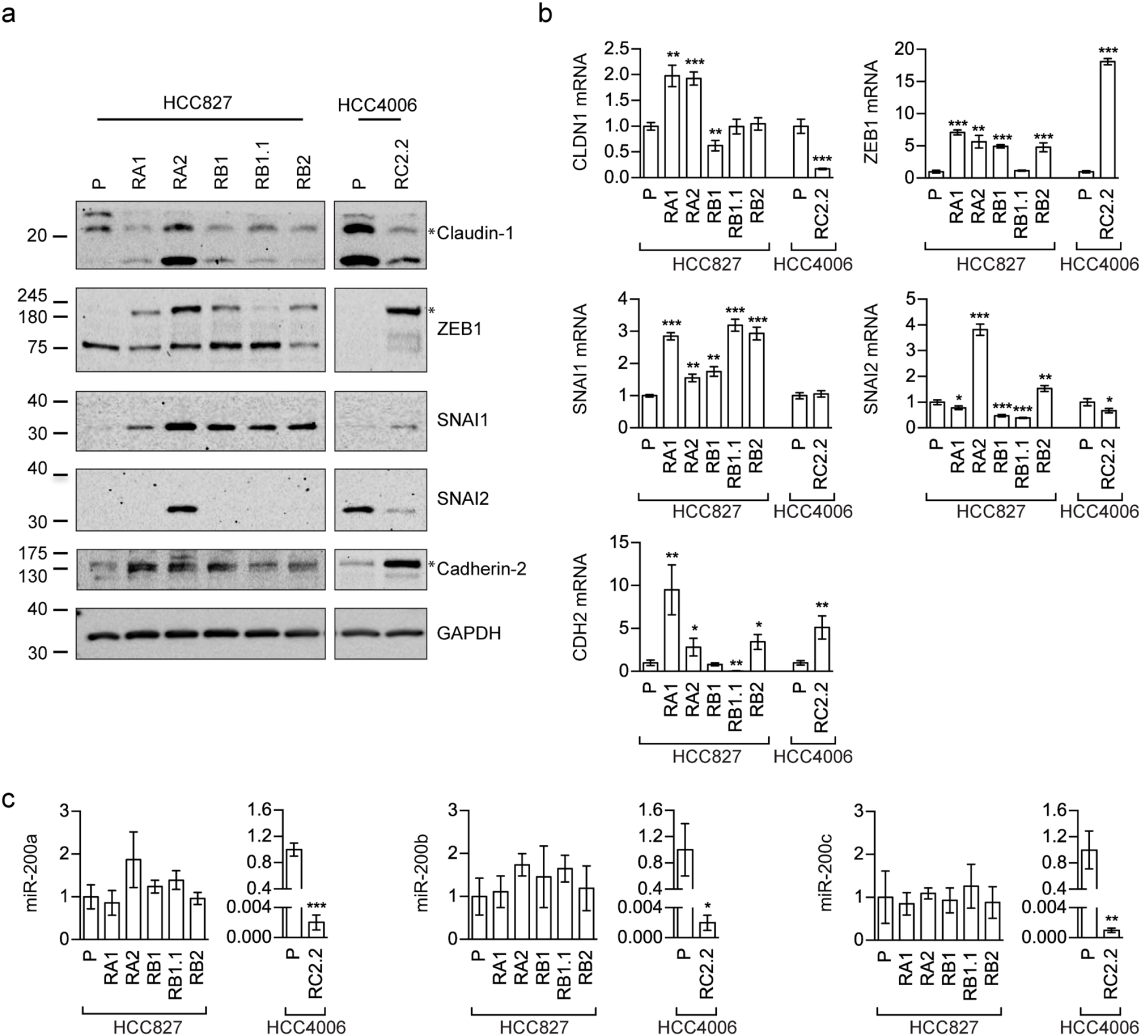
EMT markers expression in erlotinib-sensitive and -resistant cell lines **(a)** Western blot analysis of Claudin-1, Cadherin-2 (N-cadherin antibody), SNAI1 (Snail antibody), SNAI2 (Slug antibody) and ZEB1 in the indicated parental (P) and erlotinib-resistant cell lines; **(b)** qPCR analysis of the indicated mRNAsnormalized to the rp-L31 mRNA. Expression levels (y-axis) are expressed as relative abundance of derived compared to parental (P) cell lines (mean ± SD of triplicate determinations). **(c)** qPCR analysis of miR-200 family members (miR-200a, miR-200b and miR-200c) normalized to RNU24. Data are expressed as fold changes in expression levels of derived compared to parental (P) cell lines (mean ± SD of triplicate determinations). Asterisks in the figure indicate significant t-test p-values relative to the comparison of given derived cell lines with the corresponding parental one. ^*^ p < 0.05; ^**^ p < 0.01; ^***^ p < 0.001. Only significant p-values are shown.

Vimentin and Cadherin-1 despite generally used to characterize the EMT phenotype, could be not sufficient to identify and describe complex EMT features. Therefore, the expression levels of other EMT-related markers, such as Cadherin-2 (also known as N-cadherin), Claudin-1 (CLDN1), SNAI1 (also known as Snail), SNAI2 (also known as Slug) and ZEB1, were also investigated by western blotting (Figure 3a) and qPCR analysis (Figure 3b). ZEB1 and SNAI1 resulted upregulated in all erlotinib-resistant cell lines, as expected for an EMT phenotype [3, 32]. Because EMT is regulated, among others, by miR-200 family targeting *ZEB* [33–35], we also investigated the expression status of some miR-200 family members by qRT-PCR. Remarkably, miR-200a, miR-200b, and miR-200c were highly downregulated only in the HCC4006 erlotinib-resistant cell line, RC2.2, while their expression levels in all HCC827 erlotinib-resistant cell lines were comparable to parental cell line expression levels (Figure 3c).

A more complex picture was found for some EMT markers. In particular Cadherin-2 and Claudin-1 are observed respectively upregulated and downregulated in the HCC4006-derived RC2.2 cell line while in the HCC827-derived cell lines, Cadherin-2 appears to be upregulated only in RA1, RA2 and RB1 cell lines (Figure 3a) and Claudin-1 is downregulated in all cell lines with the exception of RA2 (Figure 3a) at the protein level. At the mRNA level a significant increase of Claudin-1 (1 fold) was observed in both RA1 and RA2 cell lines (Figure 3b). SNAI2 is downregulated in the RC2.2 cell line and barely detectable, at the protein level, in all HCC827-derived erlotinib-resistant cell lines, with the exception of RA2 (Figure 3a and 3b).

Overall the data suggest that the total population of the RC2.2 cell line, that does not express Cadherin-1 and expresses high levels of Vimentin, is in a mesenchymal state while the majority of HCC827-derived erlotinib-resistant cell lines might likely contain subpopulations with intermediate states of EMT.

### Cell migration features of erlotinib-resistant NSCLC cell lines

**Figure 4:**
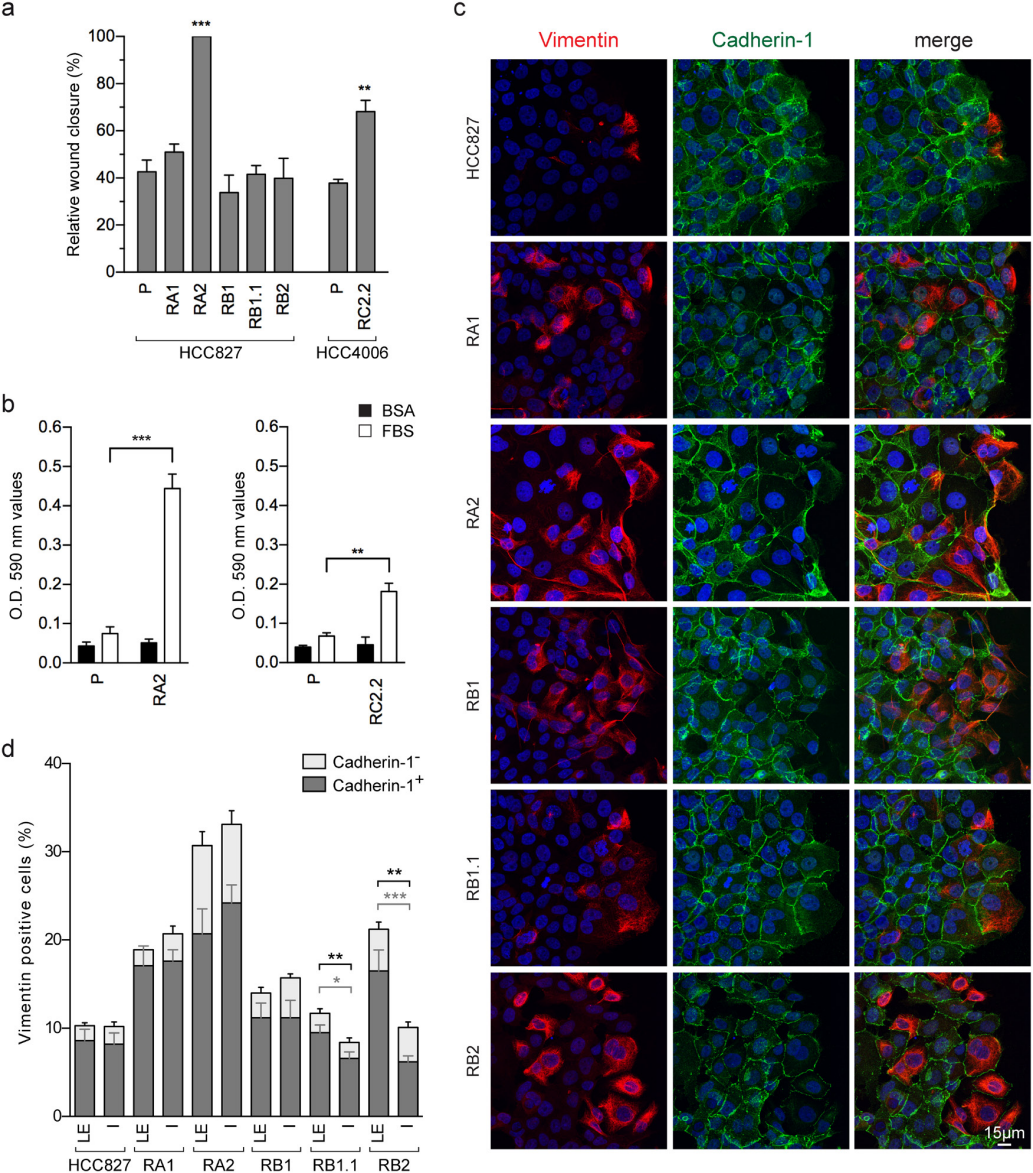
Cell migration of erlotinib-sensitive and erlotinib-resistant NSCLC cell lines **(a)** Analysis of cell migration by wound healing assays. The wound closure was quantified for each of the indicated cell lines at 16-24h post-wound by measuring the cell-free area using the ImageJ software [64]. Bar plots represent the percentage of relative wound closure (mean ± SEM), calculated as described in material and methods. Data were obtained by the analysis of 5-10 immunofluorescence images for each cell line at the time points indicated and they are representative of 3 independent experiments. **(b)** Analysis of transwell migration assays. Bar plots represent the optical reading (OD) (mean ± SEM), of BSA (black) and FBS (white) guided transwell cell migration assays as described in material and methods. The data are representative of 3 independent experiments. Asterisks in the figure indicate significant t-test p-values relative to the comparison of given derived cell lines with the corresponding parental one. ^**^ p < 0.01; ^***^ p < 0.001. Only significant p-values are shown. **(c)** Confocal microscopy images of HCC827 and derived erlotinib-resistant cell lines stained with specific reactive antibodies for Cadherin-1 (green), Vimentin (red) and Hoechst 33258 for nuclei (blue) at the wound edge. Images represent the projection of 12-18 selected focal planes spanning 4.8-7.2 μm of the specimens. Scale bar is indicated. **(d)** Analysis of Vimentin and Cadherin-1 positive cell counts at the leading edge (LE) and inner side (IS) in wound healing assays. Stacked bars, show the percentages of all Vimentin positive cells, composed of Vimentin/Cadherin-1 double positive cells (mean± SEM) in dark gray, and Vimentin positive/Cadherin-1 negative cells (mean± SEM) in light gray. Data representative of 1-2 independent experiments were obtained by cell counts at the leading edge and inner side of wound healing assays of 9-12 immunofluorescence images of 0.065 mm^2^. In the inner side, lumen or empty areas were discarded. The cells in the wounds were not counted. Only significant p-values are shown, in gray for double positive cells and in black for total Vimentin positive cells. ^*^ p < 0.05; ^**^ p < 0.01; ^***^ p < 0.001.

We used wound-healing assays in the erlotinib-resistant cell lines in order to investigate the effect of the observed EMT phenotypes on cell migration. The RC2.2 (HCC4006-derived) and the RA2 (HCC827-derived) erlotinib-resistant cell lines show an increased rate of wound closure, while for all other erlotinib-resistant cell lines quantitative differences in terms of wound closure capacity were not observed by comparison with their parental cell line (Figure 4a). Importantly, the RA2 and RC2.2 cell lines increased cell migration was also confirmed in transwell cell migration assays (Figure 4b).

Many cancer cells are known to exhibit collective invasion potential wherein cell–cell adhesions remain intact. Accordingly, in EMT phenotypes, marked by an heterogeneous cancer cell population with epithelial, E/M and mesenchymal features, the cells with intermediate E/M phenotypes could potentially contribute to a collective cell migration due to their ability to interact with epithelial cells by cell-cell contacts while also being migration-prone for their mesenchymal features. Indeed, in the wound healing assays of the HCC827-derived erlotinib-resistant cell lines, E/M Vimentin and Cadherin-1 double positive cells are detected at the wound leading edge in cell-cell contact with Cadherin-1 positive and Vimentin negative cells (Figure 4c). In particular, the RB1.1 and RB2 cell lines showed a clear increase in the number of E/M double positive cells at the leading edge compared with the number of the ones located in the inner side (Figure 4d). Differentially, the mesenchymal RC2.2 cell line was characterized by the presence, at the wound leading edge, of small groups of associated Vimentin and Cadherin-2 positive cells with apparently looser adherens junctions. These features are reminiscent of the ones described in migrating mesodermal cells during embryonic development [36, 37] (Supplementary Figure 2).

### Cell invasion and sphere-forming abilities of erlotinib-resistant NSCLC cell lines

**Figure 5:**
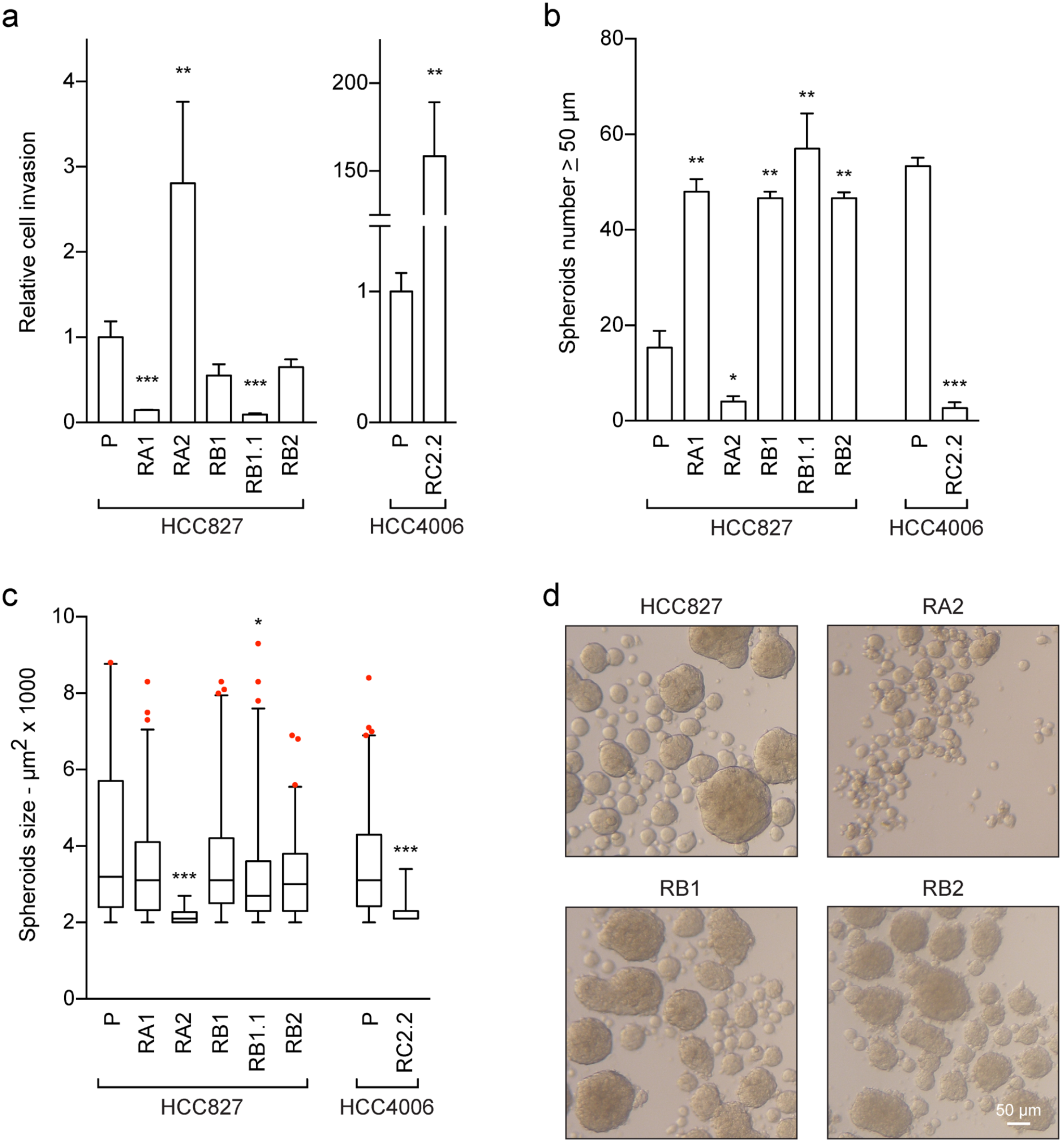
Cell invasion and stem-like features of erlotinib-sensitive and -resistant NSCLC cell lines **(a)** Analysis of cell invasion. The cell invasion ability of the indicated NSCLC cell lines was assayed using matrigel-coated transwells as described in material and methods. In three independent experiments the samples were assayed in triplicate. The area of the membrane filters occupied by successfully invasive cells was quantified 24 h after plating by ImageJ software. Data are represented as relative fold change ± SEM of parental cell lines. ^**^ p < 0.01; ^***^ p < 0.001. **(b-d)** Sphere-forming assays. The number and surface area of spheroids with a diameter ≥50 μm were recorded and quantified by ImageJ software 6-7 days after the start of the culture period. Bar plots represent in (b) the spheroids number and in (c) the spheroids size. The data are representative of 2-3 independent experiments. Asterisks in the figure indicate significant p-values of comparison of derived cell lines vs the corresponding parental one. ^**^ p < 0.01; ^***^ p < 0.001. Only significant p-values are shown; (d) representative images of spheroids are shown, scale bar is indicated. Statistical analysis of invasion and spheroids data was performed using the non-parametric Mann-Whitney U test. Differences between the means were considered statistically significant when p≤ 0.05.

Erlotinib-resistance and EMT phenotypes can associate with more aggressive behaviours, such as increased cell invasion and stem-like features. Therefore to further study the functional phenotype of the erlotinib-resistant NSCLC cell lines, matrigel-coated transwell invasion and sphere-forming assays were performed. The matrigel assays showed an increased cell invasion of the RA2 and RC2.2 NSCLC cell lines when compared to their parental erlotinib-sensitive cell lines (Figure 5a and Supplementary Figure 3). Instead similar or decreased invasion ability was recorded for the RA1, RB1, RB1.1 and RB2 cell lines (Figure 5a and Supplementary Figure 3).

Regarding the sphere-forming assays, we observed that the number of spheroids of the RA1, RB1, RB1.1 and RB2 cell lines with a diameter ≥ 50 μm was 2-3 fold higher than of the HCC827 parental cell line (Figure 5b) while the spheroid size was similar (Figure 5c, 5d). On the contrary the number of spheroids of the RA2 and RC2.2 cell lines was respectively 4 and 10 fold lower than of their parental cell lines. Moreover, the spheroids of the RA2 and RC2.2 were remarkably smaller than the spheroids of the other NSCLC cell lines (Figure 5c and 5d).

Overall the results show that the increased *in vitro* cell migration and cell invasion features of the RA2 and RC2.2 erlotinib-resistant cell lines do not associate with an increased stem-like ability. On the contrary the majority of HCC827-derived erlotinib resistant NSCLC cell lines with intermediated E/M phenotypes that do not show an increase in cell migration or invasion are more prone to form spheroids *in vitro* when compared to their parental erlotinib-sensitive cell line.

### Gene expression analysis in erlotinib-resistant NSCLC cell lines

**Figure 6:**
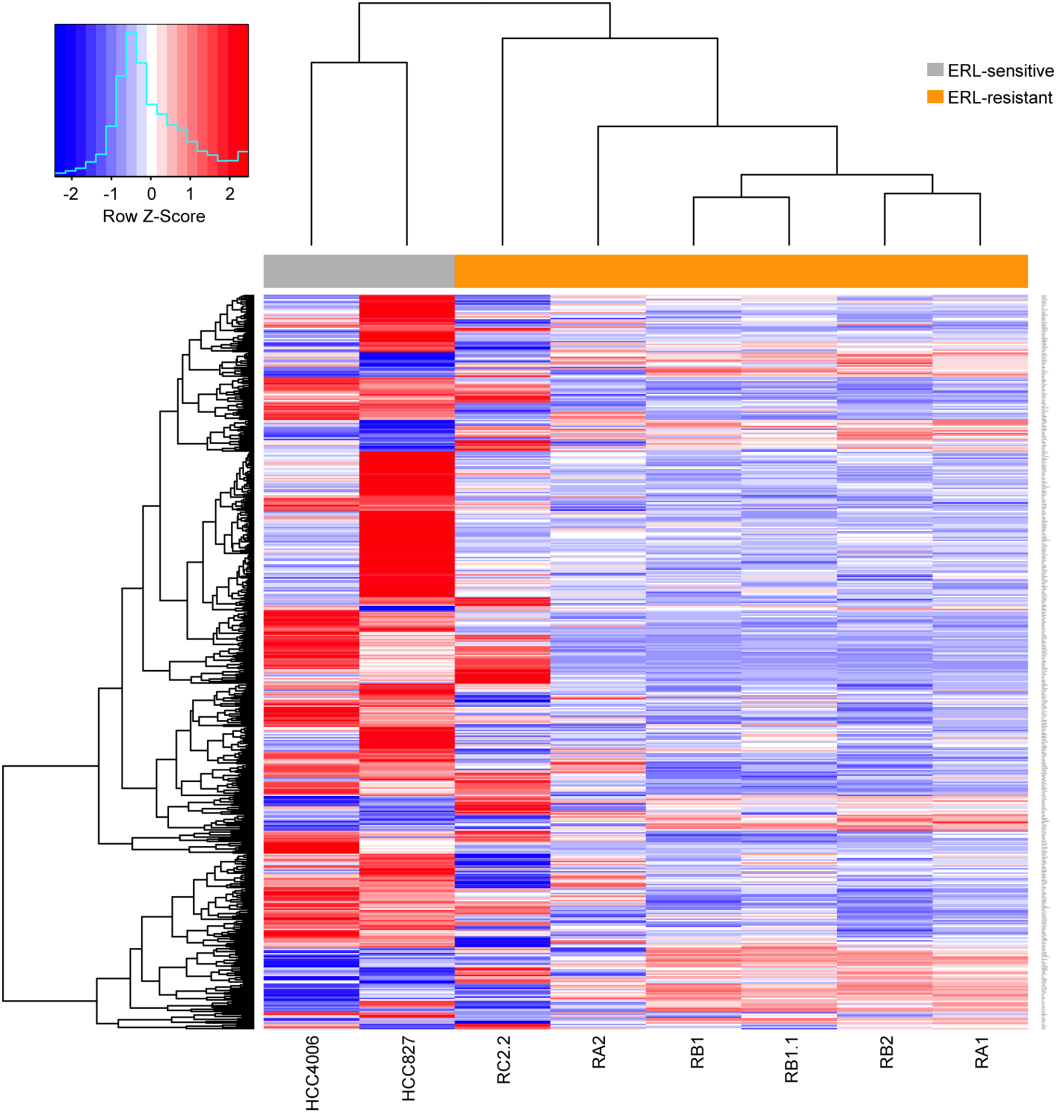
Differentially expressed genes in erlotinib-resistant NSCLC cell lines Hierarchical biclustering heatmap of expression levels for genes scored as differentially expressed in at least 5/6 comparisons of erlotinib-resistant derived cell lines to their erlotinib-sensitive parental cell lines. Data are row-scaled and rendered on a blue (lower expression) to red (higher expression) scale.

To identify genes and pathways involved in erlotinib-resistance, microarray gene expression analysis was performed. Thousands of genes differentially expressed in the erlotinib-resistant cell lines were identified (Table 1). Interestingly, a subset of these genes showed recurrent deregulation associated with erlotinib resistance. In particular, over 500 genes were found to be differentially expressed in at least 5/6 erlotinib-resistant cell lines showing recurrent upregulation (114 genes) or downregulation (393 genes) (Figure 6). Differentially expressed genes were ranked according to average log-fold changes across all the 5/6 comparison of expression levels between erlotinib-resistant cell lines and erlotinib-sensitive parental cell lines (Supplementary Table 2). While several interesting genes appear among the differentially expressed we decided to focus our analysis on the impact that differentially expressed genes have on pathways and biological processes. Remarkably, functional analysis of modulated genes identified enriched annotation to several pathways and biological processes that may be related to the EMT phenotype, such as Cadherin and TGF β signaling pathways, regulation of actin cytoskeleton and response to wounding (Table 2-3; Supplementary Table 2).

**Table 1:** Microarray gene expression analysis

	RA1	RA2	RB1	RB1.1	RB2	RC2.2
Up-regulated	841	1183	1041	740	1171	2172
Down-regulated	1091	1333	1244	1006	1560	2109

Counts of differentially expressed genes in erlotinib-resistant cell lines.

**Table 2:** Microarray gene expression analysis

Category	Term ID	Term	P-value	Count	Fold Enrichment
PANTHER_PATHWAY	P00012	**Cadherin signaling pathway**	1.5E-03	10	3.5
PANTHER_PATHWAY	P00057	**Wnt signaling pathway**	4.3E-03	14	2.3
KEGG_PATHWAY	hsa04060	Cytokine-cytokine receptor interaction	5.6E-03	12	2.6
KEGG_PATHWAY	hsa04640	Hematopoietic cell lineage	1.7E-02	6	3.9
KEGG_PATHWAY	hsa04360	Axon guidance	2.5E-02	7	3.1
KEGG_PATHWAY	hsa04350	**TGF-beta signaling pathway**	6.5E-02	5	3.2
KEGG_PATHWAY	hsa04810	**Regulation of actin cytoskeleton**	8.1E-02	8	2.1

Enriched pathways of erlotinib-resistant differentially expressed gene set.

**Table 3:** Enriched biological processes of erlotinib-resistant differentially expressed gene set

Category	Term ID	Term	P-value	Count	Fold Enrichment
GOTERM_BP_FAT	GO:0009611	**Response to wounding**	8.3E-05	25	2.5
GOTERM_BP_FAT	GO:0042127	Regulation of cell proliferation	2.5E-04	31	2.0
GOTERM_BP_FAT	GO:0045937	Positive regulation of phosphate metabolic process	6.6E-04	9	4.7
GOTERM_BP_FAT	GO:0007156	**Homophilic cell adhesion**	9.3E-04	10	4.0
GOTERM_BP_FAT	GO:0042981	Regulation of apoptosis	1.6E-03	29	1.9
GOTERM_BP_FAT	GO:0006955	Immune response	1.7E-03	26	2.0
GOTERM_BP_FAT	GO:0001932	Regulation of protein amino acid phosphorylation	1.8E-03	11	3.3
GOTERM_BP_FAT	GO:0042509	Regulation of tyrosine phosphorylation of STAT protein	2.8E-03	5	8.4
GOTERM_BP_FAT	GO:0051050	Positive regulation of transport	3.8E-03	12	2.8
GOTERM_BP_FAT	GO:0051046	Regulation of secretion	5.6E-03	11	2.8
GOTERM_BP_FAT	GO:0050865	Regulation of cell activation	6.6E-03	10	3.0
GOTERM_BP_FAT	GO:0007155	**Cell adhesion**	8.3E-03	24	1.8
GOTERM_BP_FAT	GO:0006954	Inflammatory response	9.9E-03	14	2.2
GOTERM_BP_FAT	GO:0031099	Regeneration	1.0E-02	6	4.5
GOTERM_BP_FAT	GO:0030111	**Regulation of Wnt receptor signaling pathway**	1.1E-02	5	5.7
GOTERM_BP_FAT	GO:0009967	Positive regulation of signal transduction	1.2E-02	13	2.3
GOTERM_BP_FAT	GO:0007267	Cell-cell signaling	1.2E-02	21	1.8
GOTERM_BP_FAT	GO:0050731	Positive regulation of peptidyl-tyrosine phosphorylation	1.2E-02	5	5.5
GOTERM_BP_FAT	GO:0045428	Regulation of nitric oxide biosynthetic process	1.5E-02	4	7.7
GOTERM_BP_FAT	GO:0002694	Regulation of leukocyte activation	1.5E-02	9	2.8

### Analysis of EMT-related gene expression in erlotinib-resistant NSCLC cell lines

In order to analyze the EMT phenotypes associated to erlotinib-resistance, we collected five lists of genes whose expression has been previously associated to EMT (Table 4; Supplementary Table 3) (Byers et al., 2013 [38]; Gröger et al., 2012 [39]; Huang et al., 2013 [15]; PAHS-090 [40]; R&D System [41]). These gene lists were pooled together (335 genes) and 270/335 genes (COMBO_270 gene list) also interrogated in our microarray experiments were further investigated (Supplementary Table 3). The first evidence gained from the analysis was that the above EMT-related lists showed little gene overlap among each other, with the maximum overlap (N=15) found between the Byers et al. and the Huang et al. gene lists (Supplementary Figure 4). However, hierarchical clustering based on expression levels relative to each of these EMT-related gene list alone (data not shown) yielded a grouping of the different NSCLC cell lines similar to that observed by considering the pooled list of 270 genes (Supplementary Figure 5a). In particular, the erlotinib-sensitive and -resistant NSCLC cell lines separate in the cluster primarily based on their cellular origin. This evidence was also confirmed by Principal Component Analysis (PCA) of the expression profiles of the pooled list, which shows the maximum distance in the plane identified by the first and second principal components (PCs), as well as in any other PCs plane, as the one separating the HCC827 from the HCC4006 parental cell lines (both epithelial-like), (Supplementary Figure 5b). Altogether, these results indicate that the EMT-related gene sets were not able to capture the common epithelial phenotype observed in parental cell lines. Therefore we decided to extend the analysis by applying more sensitive supervised methods to further interrogate the EMT gene lists.

**Table 4:** Description of the EMT-related gene lists

Reference	n. of genes	n. of genes analyzed
Byers et al., 2013 [36]	76	61
Huang et al., 2013 [13]	33	29
Gröger et al., 2012 [37]	131	103
PAHS-090, Qiagen [38]	84	70
R&D System [39]	98	78

### Supervised selection of EMT-related genes discriminating epithelial vs mesenchymal traits in NSCLC cell lines

**Figure 7:**
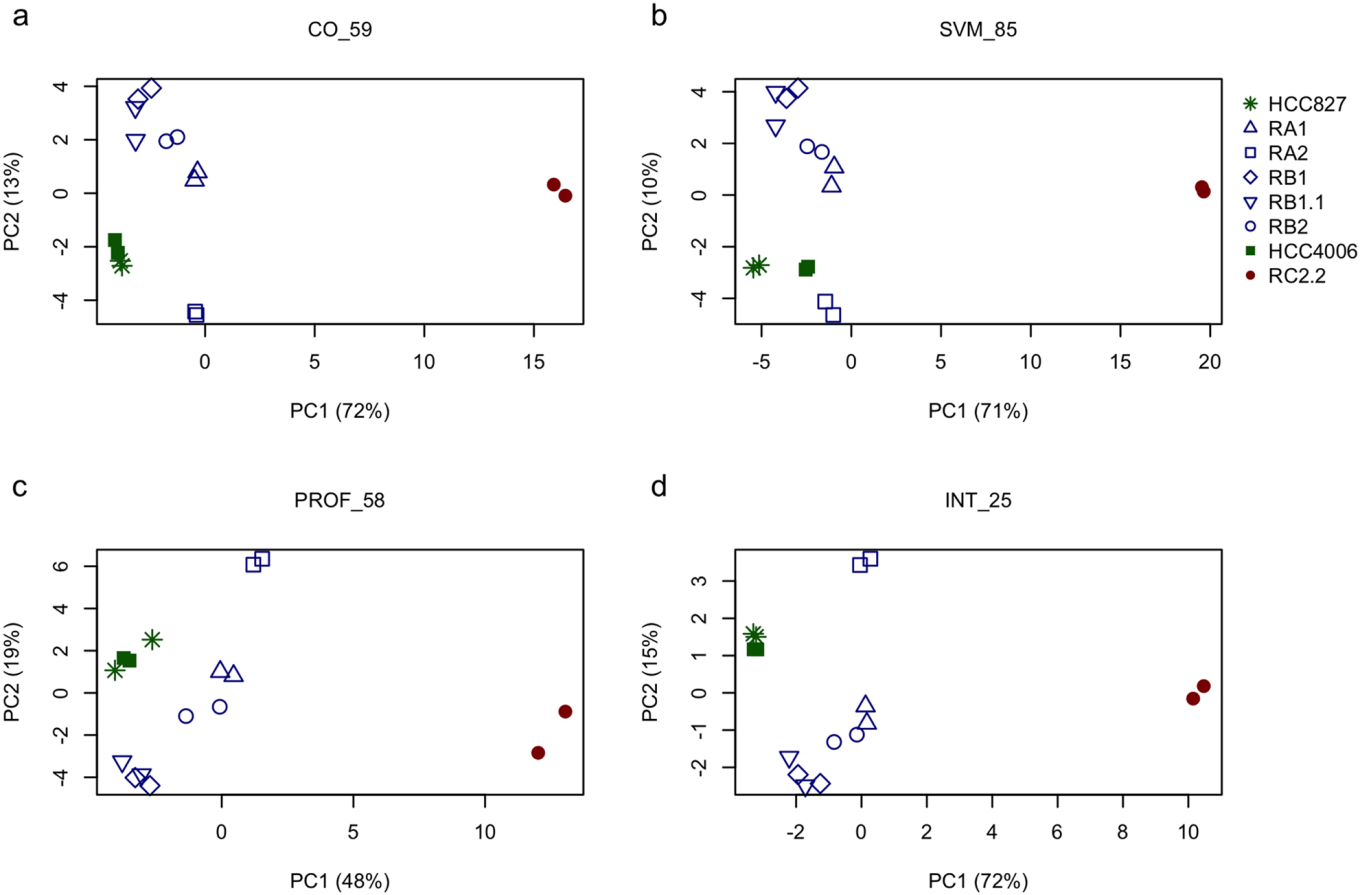
Principal components analysis of selected EMT-related gene sets Relative positioning of erlotinib-sensitive parental cell lines (HCC827 and HCC4006) and their erlotinib-resistant derived cell lines in the plane identified by principal components one and two (ie: the ones explaining most of the variation observed in the data) based on principal component analysis applied to the expression levels of differently selected subsets of EMT-related genes: **(a)** 59 EMT-related genes selected by cut-off on gene expression (CO), named *CO_59* in the figure; **(b)** 85 EMT-related genes selected by support vector machines classification (SVM), *SVM_85*; **(c)** 58 EMT-related genes selected by expression profile analysis (PROF), *PROF_58* and **(d)** 25 genes common to all the previous gene subsets, *INT_25*. Percentages indicated in brackets on the x- and y-axis represent the amount of data variation explained by the principal component one and two (PC1 and PC2), respectively. Colors indicate the EMT phenotype of the corresponding cell line: green for epithelial, blue for epithelial/mesenchymal and red for mesenchymal.

**Figure 8:**
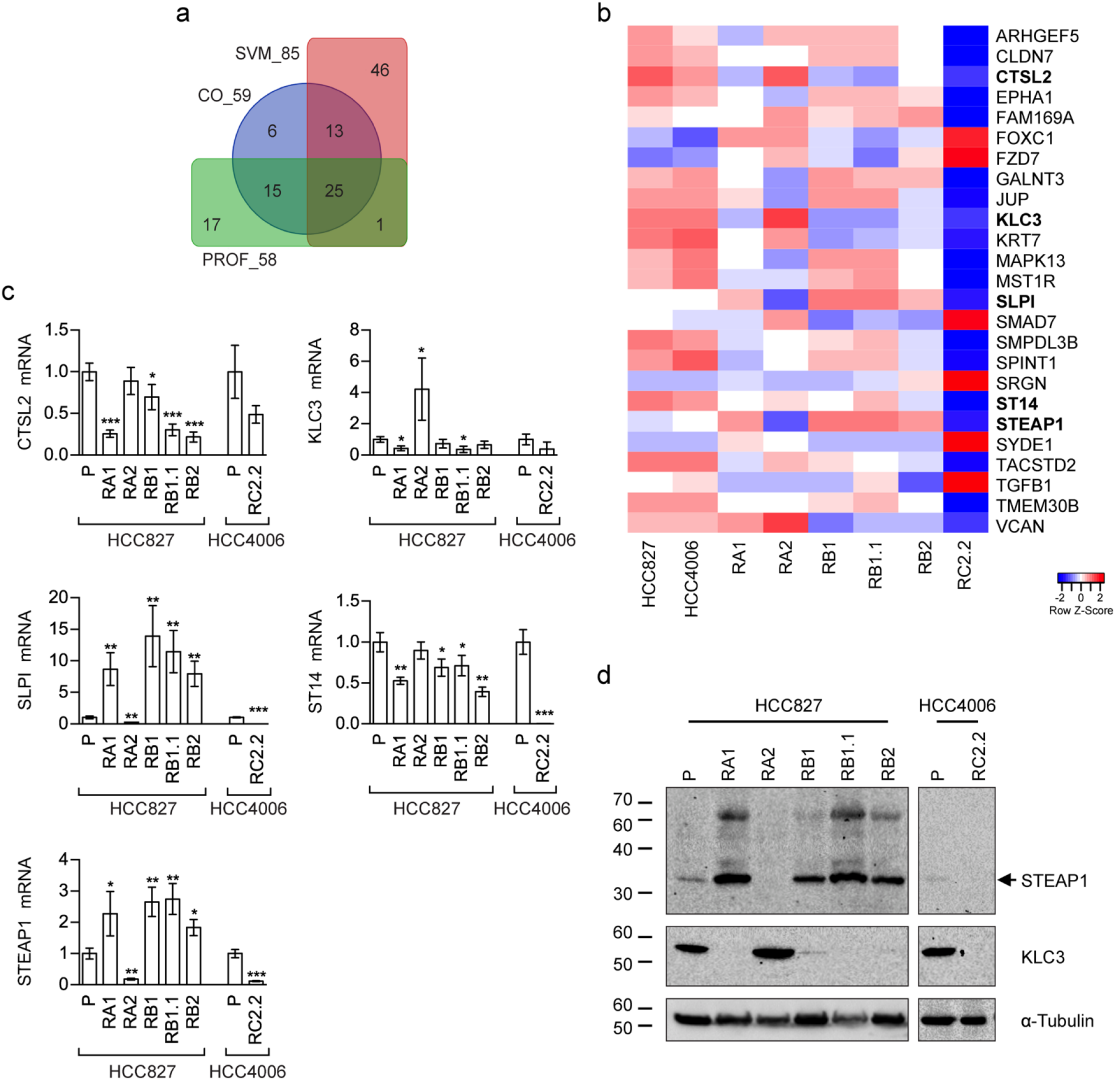
EMT-related gene subsets discriminating epithelial, mesenchymal and intermediate E/M phenotypes **(a)** Venn diagram showing overlaps among the three differently selected subsets of EMT-related genes. Abbreviations. *CO_59*: 59 EMT-related genes selected by cut-off on gene expression (CO); *SVM_85:* 85 EMT-related genes selected by support vector machines classification (SVM); *PROF_58:* 58 EMT-related genes selected by expression profile analysis (PROF). **(b)** Heatmap representing the relative expression levels across erlotinib-sensitive and erlotinib-resistant NSCLC cell lines for 25 genes shared by all the EMT-related gene subsets represented in (a). Data are row-scaled and rendered on a blue (lower expression) to red (higher expression) scale. Bold gene labels highlight the genes validated by qPCR. **(c)** qPCR analysis of CTSL2, KLC3, SLPI, ST14, and STEAP1 mRNAs normalised to rp-L31 mRNA and expressed relative to their levels in parental (P) cell lines (mean ± SD). qPCR data are representative of three independent experiments. Asterisks indicate significant t-test p-values in the comparison of a given derived cell line with the corresponding parental cell line: ^*^ p < 0.05; ^**^ p < 0.01; ^***^ p < 0.001. Only significant p-values are shown. **(d)** Western blot analysis of STEAP1 and KLC3 in the indicated parental (P) and erlotinib-resistant cell lines.

Three different supervised methods were applied to select genes maximally associated to the intermediate E/M phenotypic differences observed in the tested NSCLC cell lines: (i.) cut-off on gene expression (CO); (ii.) support vector machine (SVM); (iii.) expression profile analysis (PROF). Specifically, we used these supervised methods to identify subsets of genes having similar expression levels within the epithelial cell lines (HCC827 and HCC4006) while exhibiting different expression in the mesenchymal (RC2.2) and the cell lines with E/M features (RA1, RA2, RB1, RB1.1, RB2) (see the methods section for details). The gene subsets resulting by application of the above three methods, hereafter also referred to as *CO_59, SVM_85, and PROF_58* according to the related selection method (CO, SVM or PROF) and the number of selected genes (59, 85 and 58 respectively) (Supplementary Table 3), were extensively analyzed by PCA. PCA results from all subsets show consistent relative positioning of the erlotinib-sensitive and -resistant NSCLC cell lines (Figure 7a–7c). More in details, the HCC827-derived erlotinib-resistant cell lines appear located between the epithelial-like and mesenchymal-like cell lines, yet closer to the epithelial ones, along the first principal component (PC1). Interestingly the RB1.1 cell line, showing a small number of cells double positive for Cadherin-1 and Vimentin but high expression levels of SNAI1, groups with the other HCC827 erlotinib-resistant cells lines in the PCA plane. This result suggests that Cadherin-1 and Vimentin are likely not sufficient to identify cells with an intermediate EMT phenotype. Interestingly, the erlotinib-resistant HCC827 cell lines consistently appear sub-grouped by proximity along the second component (PC2): the RB1 with the RB1.1; the RA1 with the RB2; while the RA2 separates from the other HCC827-derived cell lines (Figure 7a–7c). Similar relative positioning in the PCA plane identified by the first and second principal components (ie: the most explicative as being the ones explaining most of the viewed differences) suggest that the three differently selected gene lists, while qualitatively diverse, are all agreeing on capturing similar cell line characteristics. In fact, these three EMT-related selected gene lists only share a limited number (=25) of genes (INT_25 list in Supplementary Table 3) as shown in the Venn diagram (Figure 8a) and the heatmap (Figure 8b). Remarkably, PCA plot relative to this restricted list of common genes recapitulates the ones obtained by analyzing the three cognate subsets of EMT-related genes (Figure 7d). Five out of the twenty-five shared EMT-related genes (namely CTSL2, KLC3, SLPI, ST14 and STEAP1) were selected for the qPCR validation in that they are expressed at different levels in the erlotinib-sensitive and -resistant cells lines in the microarray data and were previously associated to cancer development (Figure 8c). CTSL2 or cathepsin L2 gene, a pro-apoptotic target of E2F1 and a modulator of apoptosis [42] previously reported to be upregulated in several human tumors including breast and colon carcinoma [43], is downregulated in the HCC827-derived cell lines RA1, RB1, RB1.1 and RB2 as well in the HCC4006-derived RC2.2 cell line (Figure 8b, 8c). KLC3 or kinesin light chain 3, a member of the kinesin light chain gene family involved in binding cargo and regulating kinesin activity, has been reported to be upregulated in some cases of breast, ovarian, head and neck, lung, skin, urothelial and pancreatic cancers (URL: http://www.proteinatlas.org/ENSG00000104892-KLC3/cancer). KLC3 while donwregulated in the majority of our erlotinib-resistant cell lines is upregulated in the RA2 cell line (Figure 8b, 8c). Interestingly SLPI a serine protease inhibitor overexpressed in a wide variety of human cancers, including breast, lung, ovarian and colorectal carcinomas, and glioblastoma [44–48], is highly upregulated in RA1, RB1, RB1.1 and RB2 erlotinib-resistant cell lines (Figure 8b, 8c). STEAP1, a member of a family of metalloreductases described as a cell-surface antigen in prostate tissue [49, 50] and reported to be highly expressed in prostate, breast, bladder, colon ovarian carcinomas, in Ewing's sarcoma [51] and in endothelial cells in the vessels of human lung tumours [52], is highly upregulated in RA1, RB1, RB1.1 and RB2 erlotinib-resistant cell lines (Figure 8b, 8c). Finally ST14, or suppression of tumorigenicity 14, an integral membrane serine protease and a *ZEB1* responsive gene in NSCLC [31], is strongly downregulated in the RC2.2 cell line and only slightly downregulated in the majority of the HCC827-derived NSCLC cells lines (Figure 8b, 8c). The expression level of STEAP1 and KLC3 proteins was also evaluated by western blot analysis showing a profile essentially in line with the mRNA expression data (Figure 8d). STEAP1 barely detectable in HCC827 and HCC4006 erlotinib-sensitive cell lysates is strongly upregulated in RA1, RB1, RB1.1 and RB2 cell lines. KLC3 well expressed at the protein level in the parental erlotinib- sensitive cell lines is strongly downregulated in all erlotinib-resistant cell lines with the exception of the HCC827-derived RA2 cell lines (Figure 8d).

### Functional enrichment analysis of selected EMT-related gene lists

Analysis of enriched functional annotations for the three subsets of EMT-related genes highlighted pathways associated to the EMT process, including cell adhesion molecules, tight junction, Cadherin and TGF β signaling pathway. Interestingly the TGF β pathway was found enriched in all three gene lists (Tables 5-7).

**Table 5:** Enriched pathways of EMT-related genes selected by the cut-off on gene expression method (CO_59 list)

Category	Term ID	Term	P-value	Count	Fold Enrichment
KEGG_PATHWAY	hsa04514	**Cell adhesion molecules (CAMs)**	1.6E-04	6	11
BIOCARTA	h_tgfbPathway	**TGF beta signaling pathway**	4.5E-03	3	25
KEGG_PATHWAY	hsa04670	Leukocyte transendothelial migration	1.2E-02	4	8
PANTHER_PATHWAY	P00004	Alzheimer disease-presenilin pathway	1.6E-02	4	7
PANTHER_PATHWAY	P00052	**TGF-beta signaling pathway**	2.3E-02	3	6
REACTOME_PATHWAY	REACT_13552	**Integrin cell surface interactions**	3.2E-02	3	10

**Table 6:** Enriched pathways of EMT-related genes selected by the SVM method (SVM_85 list)

Category	Term ID	Term	P-value	Count	Fold Enrichment
REACTOME_PATHWAY	REACT_604	Hemostasis	1.1E-04	8	6.1
KEGG_PATHWAY	hsa04670	Leukocyte transendothelial migration	1.5E-04	7	8.2
PANTHER_PATHWAY	P00004	Alzheimer disease-presenilin pathway	2.0E-04	7	7.2
KEGG_PATHWAY	hsa04514	**Cell adhesion molecules (CAMs)**	2.8E-04	7	7.3
KEGG_PATHWAY	hsa04530	**Tight junction**	2.3E-03	6	6.2
REACTOME_PATHWAY	REACT_578	Apoptosis	4.0E-03	5	6.9
BIOCARTA	h_tgfbPathway	**TGF beta signaling pathway**	9.5E-03	3	18.1
PANTHER_PATHWAY	P00012	**Cadherin signaling pathway**	2.1E-02	5	4.3
KEGG_PATHWAY	hsa04916	Melanogenesis	3.2E-02	4	5.6

**Table 7:** Enriched pathways of EMT-related genes selected by the expression profile analysis method (PROF_58 list)

Category	Term ID	Term	P-value	Count	Fold Enrichment
PANTHER_PATHWAY	P00052	**TGF-beta signaling pathway**	1.5E-03	6	6.2
PANTHER_PATHWAY	P00005	Angiogenesis	7.7E-03	6	4.3
KEGG_PATHWAY	hsa05210	Colorectal cancer	1.1E-02	4	8.3
KEGG_PATHWAY	hsa05217	Basal cell carcinoma	3.6E-02	3	9.6
PANTHER_PATHWAY	P00004	Alzheimer disease-presenilin pathway	4.1E-02	4	4.8
KEGG_PATHWAY	hsa04621	NOD-like receptor signaling pathway	4.5E-02	3	8.5

### Effect of TGF β and TGFR-1 inhibitor on EMT features and cell growth

**Figure 9:**
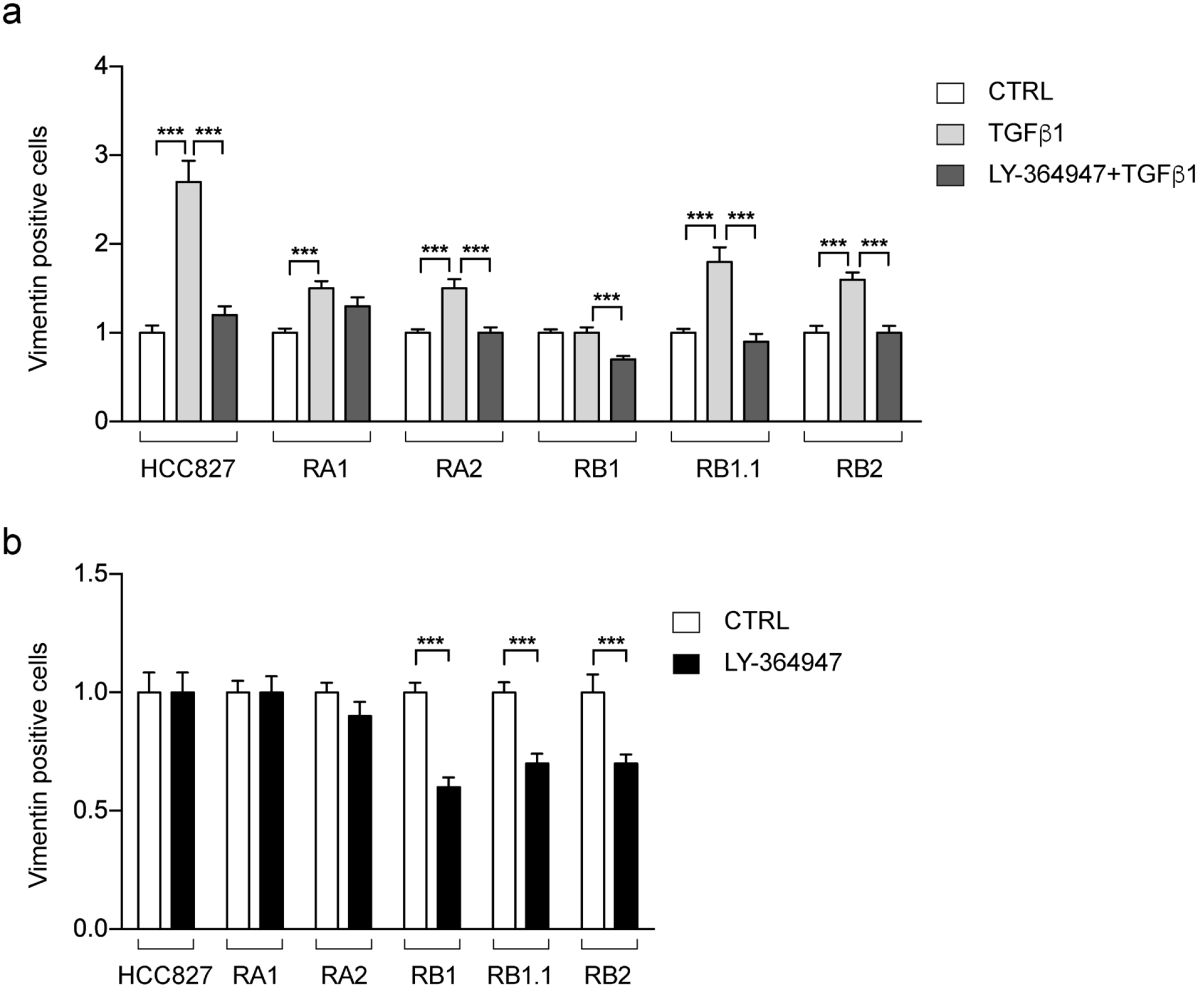
Vimentin positive cells in HCC827 erlotinib-sensitive and -resistant cell lines treated with TGF β1 and LY-364947 Bar charts represent the fold-changes (mean ± SEM) of Vimentin positive cells resulted by the comparison of cell lines treated with TGF β1 alone or in combination with LY-364947 **(a)** and LY-364947 **(b)** with samples treated only with vehicle (CTRL in a and b). Data were obtained by the analysis of 24-30 immunofluorescence images for each cell line with at least 150-200 cells/image with the exception of RC2.2 cell line in which the images contained approximately 100 cells/images. The data are resulting from at least three independent experiments. Asterisks indicate significant t-test p-values in the comparison of CTRL vs LY-364947, CTRL vs TGF β1, TGF β1 vs LY-364947+ TGF β1 are shown; ^***^ p < 0.001.

Prompted by the functional enrichment analysis, we decided to further investigate the TGF β signaling pathway. Indeed, it has been previously reported that NSCLC cells undergo EMT phenotypic changes upon short exposure to TGF β [53] or long term treatment [54] and that TGF β ligand-induced activation of the TGF β pathway led to the development of resistance to erlotinib [54, 55]. Thus, we used immunofluorescence studies to evaluate the effect of TGF β1 and a TGFR-1 inhibitor (LY-364947), alone or in combination, on Vimentin expression, as a marker of the EMT phenotype. TGF β1 treatment strongly increases the number of Vimentin positive cells in the erlotinib-sensitive parental HCC827 cell line (3 fold increase) and to a less marked extent in its derived erlotinib-resistant cell lines (≤2 fold increase) (Figure 9a). The observed increase is prevented by treatments with LY-364947 in all cell lines, except for the RA1 (Figure 9a). Remarkably, the LY-364947 treatment alone induces a 30-40% reduction in the number of Vimentin-positive cells in the RB1, RB1.1 and RB2 cell lines while not changing the number of Vimentin-positive cells in the RA1 and RA2 cell lines (Figure 9b).

Finally, growth inhibition assays indicated that LY-364947, alone or in combination with erlotinib, does not impair the viability of HCC827-derived erlotinib-resistant cell lines (Supplementary Figure 6).

Overall these results suggest that the observed EMT stable phenotypes associated to erlotinib-resistance likely appear independent or only partially mediated by TGF β-1 activation.

## DISCUSSION

Increasing evidence points to a key role played by EMT in cancer progression and drug resistance. Here we concentrated our study on the intermediate E/M phenotypes and their association to target therapy resistance in NSCLC cell lines. As observed in both physiology and pathology, a cell population can be highly heterogeneous with respect to its EMT phenotype. Indeed, we consistently observed distinct subpopulations of cells representing the three phenotypes previously described as epithelial, mesenchymal and intermediate E/M in all HCC827-derived erlotinib-resistant cell lines. Notably, the HCC827-derived cell lines studied in this work include cell subpopulations co-expressing Cadherin-1 and Vimentin, common markers for epithelial and mesenchymal phenotypes, respectively. The percentage of E/M cells was remarkably stable within each cell line, suggesting the existence of regulatory networks that preserve the cell phenotypes. It is also interesting to note that these intermediate E/M phenotypes may be selected by erlotinib treatment because the number of E/M cells was found increased in the erlotinib-resistant cell lines derived from the HCC827 cell line. On the contrary, the only established HCC4006-derived erlotinib-resistant cell line (ie: RC2.2) was found to be in a complete and stable mesenchymal state, with essentially all cells negative for Cadherin-1 and positive for Vimentin. Interestingly, only the RA2 and RC2.2 cell lines showed an increase in cell migration and cell invasion rate, suggesting that a qualitative rather than a quantitative variation of cell migration and eventually invasion may deserve further investigation in the other erlotinib-resistant cell lines. Remarkably HER3 a receptor suggested to be a strong epithelial marker and regulator of EGFR TKI sensitivity [56], was found not expressed or constitutively inactivated only in the RA2 and RC2.2 erlotinib-resistant cell lines [30].

Interestingly the increased *in vitro* cell migration and cell invasion of the RA2 and RC2.2 erlotinib-resistant cell lines do not associate with increased stem-like features. On the contrary the majority of HCC827-derived erlotinib-resistant NSCLC cell lines, with intermediated E/M phenotypes, have similar or decreased cell migration and cell invasion rate when compared with their parental cell line but show an increased sphere-forming ability. Importantly, the mRNA expression profile of some largely used stemness markers could not predict such stem-like behavior (Supplementary Table 4).

Intriguingly, while all erlotinib-resistant cells lines express higher levels of ZEB1 compared to their parental cell lines, only the RC2.2 cell line shows downregulation of miR-200a, miR-200b and miR-200c. Moreover, only the RC2.2 cell line expresses low mRNA levels of *OVOL 1, OVOL 2* and *Grainyhead-like 2 (GRHL2)* genes (Supplementary Table 2). Importantly these genes have been previously associated to the regulation of intermediate EMT phenotypes, multi-step transition between epithelial and mesenchymal states, and positive miR-200 family regulation [15, 57–60].

Furthermore, wound healing cell migration assays demonstrated a collective cell migration (reviewed in [61]) property of the HCC827-derived cell lines. The remarkable localization of Vimentin and Cadherin-1 double positive cells as well as epithelial Vimentin negative cells at the leading edge together with their collective migration highlighted the potentially dangerous contribution of E/M cell subsets in metastasis. A propensity for these E/M cell subpopulations to cluster with epithelial cells was also observed. Gene expression analysis of single epithelial, or hybrid E/M CTC clusters, could provide valuable information about their possible different functional properties.

Next, we used our NSCLC cell lines microarray data to evaluate the expression of publicly available EMT-related gene signatures and applied different supervised analytical methods for selecting novel gene sets able to partition our cancer cell lines according to their observed EMT phenotypes. Notably, despite the heterogeneous composition, all the selected gene sets showed significant enrichment of the TGF β signaling pathway, confirming the relevance of this pathway with respect to the EMT phenotypes [53–55].

Finally we investigated the effect of the modulation of the TGF β1 signaling pathway on cell survival and EMT phenotypes. We observed that the inhibition of TGFR-1, alone or in combination with EGFR inhibition, did not affect NSCLC cell lines survival, but it was able to partially decrease the number of Vimentin positive cells in some HCC827-derived erlotinib-resistant cell lines. Further investigation of the TGF β1 and of other enriched signaling pathways (i.e. the Wnt pathways) will be required to understand their contribution to the erlotinib resistance and the intermediate E/M phenotypes in our cellular model system.

Overall these results confirmed cellular heterogeneity during EMT in cancer cell lines upon drug treatment. Importantly, our findings highlighted the association an intermediate E/M phenotypes to resistance to EGFR TKI in NSCLC cell lines. Treatment of NSCLC patients sensitive or resistant to EGFR inhibitors should attempt to impact not only oncogene-driven cancer cell growth but also the EMT phenotype in early NSCLC development stages.

## MATERIALS AND METHODS

### Cells, antibodies, and reagents

The human cell lines: HCC827 (ATCC CRL-2868) and HCC4006 (ATCC CRL-2871), kindly provided by Oreste Segatto, and the erlotinib-resistant cell lines (RA1, RA2, RB1, RB1.1, RB2 and RC2.2), previously described [30] were cultured in RPMI 1640 medium (BioWhittaker, Lonza, USA) supplemented with 10 mM Hepes pH 6.98-7.30, 1 mM L-glutamine, 100 U/ml penicillin/streptomycin (BioWittaker, Lonza) and heat inactivated 10% fetal bovine serum (FBS) (Sigma-Aldrich). All cells were cultured at 37°C in a 5% CO_2_ humidified incubator. For the treatment with human transforming growth factor beta-1 (TGF β-1) (Sigma-Aldrich) (2.5 ng/ml) and LY-364947 (Sigma-Aldrich), a TGF β receptor type-1 (TGFR-1) inhibitor (1 μM), cells were cultured in Opti-MEM reduced serum media (Thermo Fisher Scientific) for 48 hours. For the sphere assays the cells were cultured in DMEM/F12 (Gibco, Grand Island, NY, USA) supplemented with commercial hormone mix B27 (Gibco), EGF (20 ng/mL; Gibco), FGF (20 ng/mL), purchased from ThermoFisher Scientific, and heparin (10U/mL) (Sigma-Aldrich). The primary antibodies used Vimentin (D21H3), E-cadherin (24E10), N-cadherin (D4R1H), Claudin-1 (D5H1D), Snail (C15D3), Slug (C19G7), ZEB1 (also known as TCF8, D80D3), GAPDH (D16H11) were from Cell Signaling Technology; Vimentin (V9 and V9Cy3), α-Tubulin (DM1A), β-actin (AC15) and horseradish peroxidase (HRP)-β-actin (AC15) were purchased from Sigma-Aldrich; GAPDH (clone 1D4) was obtained from Novus Biologicals; Phalloidin Alexa Fluor-488 and E-cadherin (HECD-1) were from Thermo Fisher Scientific; N-Cadherin (32/N-Cadherin), E-cadherin (CD324)-Alexa Fluor-647, Vimentin Alexa Fluor-488 and isotype control antibodies: Alexa Fluor-647 Mouse IgG1k (Clone MOPC-21), Alexa Fluor-488 Mouse IgG1k (Clone MOPC-21) were from BD Biosciences; STEAP (B-4) and KLC3 (E-7) were from Santa Cruz Biotechnology. Secondary antibodies goat anti-rabbit IgG (H+L)-HRP and goat anti-mouse IgG (H+L)-HRP were purchased from Bio-Rad; goat anti-mouse Alexa Fluor-488 and 546 and Hoechst 33258 were obtained from Thermo Fisher Scientific. Erlotinib hydrochloride salt, an EGFR tyrosine kinase inhibitor, was from LC Laboratories, USA; Corning matrigel basement membrane matrix growth factor reduced was from BD Biosciences. Stock solutions (10 mM) for all inhibitors were prepared in DMSO and stored at -20°C or -80°C as suggested by the product datasheet. MTT, 3-(4,5-methylthiazol-2-yl)-2,5-diphenyltetrazolium bromide and bovine serum albumin were from Sigma-Aldrich. MTT stock solution (5 mg/mL in H_2_O, sterilized by filtration) was stored at 4°C for 1 month.

Power SYBR Green PCR master mix was from Applied Biosystems. Trizol reagent was from Life Technologies, the GoScript reverse transcription system kit was from Promega.

### Cell growth inhibition MTT assay

MTT assay was performed as previously described [30]. Briefly cells (1-2 x10^4^ cells/well) plated in 96-well plates at day 0 were treated at day 1 with increasing concentrations of erlotinib or LY-364947 (from 64 pM to 10 μM) or their combination in the ratio (1:3) in complete tissue culture medium and cultured for 72 hours at 37°C in 5% CO_2_. Next, cells were gently washed with 1x PBS, incubated for 4 hours with MTT and processed for color detection with DMSO. The resulting purple solution was spectrophotometrically measured at 570 nm and 630 nm [62, 63].

### Wound healing assay

The HCC827 and HCC4006 parental cell lines and the erlotinib-resistant derived cell lines were plated onto 35 mm plastic petri dishes and grown until confluence. A cell-free area was introduced in a monolayer of cells using a sterile yellow tip (time *t*_*0*_) and then cells were allowed to migrate from the edge of the scratch at 37°C/5% CO_2_. The monolayer was imaged at different time points to record the size of the wound closure at 5-24 (time *t*) hours with an Olympus BX41 microscope using a 4-10x lens and 8-10 images were recorded with an Olympus SP-350 camera. The area of wound coverage was calculated using the ImageJ software [64]. The degree of cell migration was expressed as the percentage of relative wound closure calculated as:

$$\frac{Area\;t_{0}-Area\;t}{Area\;t_{0}}*100$$

For immunofluorescence and confocal microscopy analysis the cells were also subjected to wound healing assay with the μ-Dish^35mm^ with Culture Insert (Ibidi) and an equal number of cells (0.5-1 × 10^5^) in 70 μl of culture medium was seeded into the two reservoirs of the same insert and incubated at 37°C/5% CO_2_. After appropriate cell attachment (24 h) the culture insert was gently removed using sterile tweezers, and a 500 μm wide cell-free gap (wound) was generated. The dish was then filled with 2 mL of FBS containing medium and incubated for 8-16 hours. Then cells were processed for immunofluorescence and confocal microscopy analysis.

### Transwell migration assay

Assays were performed using a transwell (Corning Costar, Rochester, NY, USA) containing a polycarbonate membrane filter (8μm pore size) for 24-well plates according to the manufacturer’s instructions. HCC827 and RA2 (1x10^5^), HCC4006 (2.5x10^4^) or RC2.2 (2.5x10^4^) were seeded onto the upper surface of a filter with a volume of 100 μl RPMI 1640 medium containing 0.5% BSA and placed into the lower wells containing 600 μl of complete medium with 10% FBS or 0.1% BSA (negative control) to induce cell migration. The migration transwell chambers were incubated for 16h at 37°C in a humidified atmosphere containing 5% CO2. Following incubation, cells were fixed in 3.7% PFA for 10 min at room temperature, stained with 0.1% crystal violet (20% methanol solution) for 10 min, then washed 3 times with PBS. Next, the cells on the upper side of the membrane were removed by cotton swabs, and the crystal violet staining on the bottom surface of the membrane was eluted in 10% acetic acid and read at an optical density of 590 nm with a Varioskan Lux instrument.

### Transwell invasion assay

Matrigel invasion assays using transwell chambers (Corning Costar, Rochester, NY, USA) containing a polycarbonate membrane filter (8μm pore size) for 24-well plates coated with matrigel was used to determine NSCLC cell invasion properties. In brief, 1 mg/ml matrigel (50 μl) was added to each insert and solidified in a 37°C incubator for 5 h to form a thin gel layer. Next, 24 h serum-starved cells (3×10^5^/w) in RPMI 1640 containing 0.5% BSA were seeded in the upper chamber, and 500 μl RPMI 1640 containing 10% FBS was added to the lower chamber. After 24 h incubation at 37°C in a 5% CO_2_ atmosphere, cells that remained in the upper chamber were removed by cotton swabs and penetrating cells were stained with 1% crystal violet in 20% methanol. Then, the membranes were rinsed several times with water and images were recorded using a stereomicroscope Leica MZ12 served by an Olympus SP-350 camera. Next images were processed with the Image software in order to calculate the area of the membrane filter occupied by invasive cells.

### Sphere forming assay

The assay was performed essentially as previously described [65]. Briefly single-cell suspension cultures were prepared at densities of 5x10^3^ cells per well in serum-free medium DMEM/F12 supplemented with B27 supplement, EGF (20 ng/mL), FGF (20 ng/mL), and heparin (10 U/mL), and seeded into 6-well ultra- low attachment plates (Corning, Corning, NY, USA). Culture medium was replaced every 3 days; the number and size of the spheroids were recorded at 6-7 days after the start of the culture period using a stereomicroscope Leica MZ12 served by an Olympus SP-350 camera. The number of spheroids exceeding a diameter of 50 μm and their surface area were evaluated by the ImageJ software [64].

### Determination of IC_50_ value and regression trendline

Data were analysed by GraphPad Prism software v6.0c. The optical density values of cells treated with drugs, obtained by MTT assay reading, were expressed as percentage of cell survival and normalized with the value of cells treated with vehicle (DMSO). The regression trendline were fitted using a non-linear regression method and IC_50_ values were determined using a sigmoidal dose response inhibition variable slope method. The synergy of erlotinib and LY-364947 was evaluated by the Chou-Talalay method [66] using CompuSyn software [67] as previously described [30].

### Immunofluorescence and confocal microscopy analysis

HCC827 parental and erlotinib-resistant cells, grown on poly-L-lysine coated coverslips or on 35 mm tissue culture plates or on the Ibidi culture dish and HCC4006 and RC2.2 cells, on 35 mm tissue culture plates, were fixed with 3.7% PFA for 10 minutes at room temperature. Next cells were permeabilized (1x PBS, 0.1% Triton X100) for 6 minutes at room temperature, treated with blocking solution (1x PBS, 1% BSA) and stained with primary antibodies in blocking solution, for 1 hour, washed with 1x PBS for 10 times and stained with fluorochrome-labeled secondary antibodies for 45 min at room temperature and Hoechst 33258 for 5 min. HCC827 samples were analysed with laser scanning confocal microscope, TCS SP5 (Leica Microsystems, Mannheim) using a 63x (NA=1.4) or 40x (NA=1.25) oil-immersion lens with optical pinhole at 1AU. For fluorescence images Argon ion laser at 488 nm, HeNe ion laser at 543 nm and blue diode laser at 405 nm were used as excitation sources. Confocal Z-stacks were collected at 0.4 μm intervals to a total optical depth of 8-12 μm. Confocal images (at least 10 images for each sample in three independent experiments) were processed with Volocity (Improvision, Perkin Elmer) and Adobe Photoshop CS4 software for image rendering and representation of x/y view. Images for direct comparison were all collected under same parameters and representative images were chosen. HCC4006 and RC2.2 cells, stained in tissue culture plates, were analyzed with an Olympus AX70 fluorescence microscope using a 20x or 40x lens and images were recorded with an Olympus XM10 camera and processed using Olympus CellSens Standard 1.8.1 software. The following setting were used for fluorescence microscopy images acquisition: exposure time at 500 ms, gain at 4 dB, and thresholds of the fluorescence intensity on dynamic range in order to include the overall histogram area. Next, the immunoreactivity for Vimentin, was independently evaluated by two investigators and Vimentin positive cells were independently counted. When discrepancies in counting occurred, a consensus interpretation was reached after reexamination. All cell counts were carried out using the ImageJ software [64]. All image panels presented were assembled using Adobe Illustrator CS4 (14.0.0)

### Flow cytometry analysis

Adherent cells were detached from tissue cell culture plates by treatment with a cell dissociation buffer enzyme-free PBS (Thermo Fisher) at 37°C. Cells were collected in 15 ml tubes, washed with ice-cold RPMI 1640 medium supplemented with 10% FBS and centrifuged at 400g for 5 min at 4°C. Next cell pellets were first washed with ice-cold PBS supplemented with 1% FBS to block unspecific antibody binding and fixed with PFA (3.7%) for 15 min on ice. After a wash with ice-cold PBS supplemented with 1% FBS, cells were permeabilized with 0.1% Triton X-100 in PBS for 15 min at room temperature and washed with PBS+1%FBS. Then 0.5-1x10^6^ cells were stained in 5ml round bottom tubes (BD Falcon) with Alexa Fluor-conjugated antibodies for 45 min at room temperature. Cells were then washed twice with PBS+1% FBS. Finally samples were analyzed using a BD FACS Calibur flow cytometer (Becton Dickinson). Unspecific background of individual channels was determined with isotype matched controls. Plots were generated using FlowJo v10.2 software.

### Western blot analysis

Cells were lysed in ice-cold RIPA buffer (50 mM Tris-HCl pH 7.5, 150 mM NaCl, 1% NP40, 0.25% sodium deoxycholate, 1 mM EDTA) containing a combination of protease and phosphatase inhibitor cocktails or in SDS Laemmli sample buffer. Cell lysates (RIPA buffer) were quantified for proteins content with the Bio-Rad DC Protein Assay kit. Proteins (25-50 μg) were then separated by 8-12% SDS-PAGE and transferred to nitrocellulose membranes 0.45 μm (GE Healthcare Life Sciences). The membranes were blocked with 5% non-fat milk (EuroClone) or 2% BSA (Sigma-Aldrich) in 1x TBS pH 7.6-8.0 containing 0.1 or 0.2% Tween 20 (TBST) for 2 hour at room temperature (RT) and subsequently probed with primary antibodies in 5% non-fat milk or 2-5% BSA in TBST overnight at 4°C. Then membranes were washed 10 minutes for 3 times with TBST and probed with horseradish peroxidase-conjugated secondary antibodies in 5% non-fat milk in TBST for 1 hour at RT. Chemidoc XRS Bio-Rad was used for images acquisition with a chemi-luminescent camera, band signals were quantified using ImageLab 4.0 Bio-Rad software.

### RNA analysis

For mRNA analysis, total RNA was extracted using the TRIzol (Life Techologies) reagent and retro-transcribed with the GoScript Reverse Transcription System (Promega) using oligo (dT) and random primers. qPCR analysis was performed with specific primers (Table 8) and analyzed as previously described [30]. qPCR analysis was performed using a 7500 Fast Real-Time PCR System (Applied Biosystems). *Ribosomal protein L31* (*rp-L31*) was used as a reference gene to normalize quantitation of target genes for differences in the amount of total RNA in each sample.

**Table 8:** qPCR primers

qPCR Primers	Sequence 5’ > 3’
hVIM F	ACACCCTGCAATCTTTCAGACA
hVIM R	GATTCCACTTTGCGTTCAAGGT
hCDH1 F	ATTCTGATTCTGCTGCTCTTG
hCDH1 R	AGTCCTGGTCCTCTTCTC
hZEB1 F	GATGATGAATGCGAGTCAGATGC
hZEB1 R	CTGGTCCTCTTCAGGTGCC
hSNAI1 F	GCGAGCTGCAGGACTCTAAT
hSNAI1 R	GGACAGAGTCCCAGATGAGC
hSNAI 2 F	CTGGGCTGGCCAAACATAAG
hSNAI2 R	CCTTGTCACAGTATTTACAGCTGAAAG
hCLDN1 F	TGCGATATTTCTTCTTGCAGGT
hCLDN1 R	TTCGTACCTGGCATTGACTGG
hCDH2 F	GTGCATGAAGGACAGCCTCT
hCDH2 R	CCACCTTAAAATCTGCAGGC
hSTEAP1 F	TACTGGGCACAATACACGCA
hSTEAP1 R	TCCTCAAGCATGGCAGGAAT
hCTSL2 F	ACTGCAGCAGCAAAAACCTG
hCTSL2 R	ACCCCAGCTGTTTTTGACGA
hSLPI F	ACCCCAAACCCAACAAGGAG
hSLPI R	ACGCAGGATTTCCCACACAT
hKCL3 F	CGAGGAAGTGAGAAGCTGGT
hKCL3 R	CAGGAGCCCTTGGAGCATC
hST14 F	CAACCAGCATGTGAAGGTGC
hST14 R	CCGCAGTATTTCTCCCCGTT
hRPL31 F	CATCCATGGAGTGGGCTTCA
hRPL31 R	AGCTTTGTTGAGCCTGGTGT

microRNA levels were analyzed from total RNA using the following TaqMan MicroRNA Assays: miR-200a ID:000502, miR-200b ID:002251, miR-200c ID:002300 and RNU24 ID:001001(Applied Biosystems, Thermo Scientific). Relative expression was calculated using the comparative Ct method. The small nucleolar RNA U24 (RNU24) was used as a reference gene.

Both for mRNA and microRNA analysis, total RNA of HCC827 and HCC4006 parental cell lines were used as calibrator samples, relative to which differences in the RNA amount of resistant cell lines have been calculated. The data were analyzed using SDS (Ver. 1.4) software (Applied Biosystems).

### Microarray data description and analysis

For microarray gene expression analysis, total RNA was isolated using a total RNA purification kit (Norgen Biotek, Thorold, ON, Canada). RNA quantity was determined by absorbance at 260 nm using a NanoDrop ND-1000 UV-VIS spectrophotometer (Thermo Fisher Scientific, Wilmington, DE, USA). RNA quality was assessed with Agilent Bioanalyzer RNA 6000 Nano kit; 200 ng of RNA were labeled with LowInput QuickAmp Labeling Kit One-Color (Agilent Technologies), purified and hybridized overnight onto an Agilent SurePrint G3 Human Gene Expression Array (8x60K v2 G4851B - 039494) before detection, according to the manufacturer’s instructions. Agilent DNA Microarray scanner was used for slide acquisition and spot analysis was performed with Feature Extraction software (Agilent Technologies).

Microarray raw data, provided by the European Brain Research Institute (EBRI, Rome) were background corrected, log2 transformed and quantile normalised by using *limma* package [68]. A total of 42,898 probes (27,841 unique official gene symbol identifiers) were selected after filtering out control and low intensity probes. Probes that map on the same gene were summarized into one averaged expression value.

The whole Agilent microarray dataset used in this study is publicly available from the NCBI's Gene Expression Omnibus (GEO) database [69] under accession number GSE80344 at the following URL: http://www.ncbi.nlm.nih.gov/geo/query/acc.cgi?acc=GSE80344.

PCA of cell lines was performed using gene expression values as variables that were scaled to have unit variance before the analysis took place. Unsupervised hierarchical clustering was performed using the complete linkage method and Euclidean distances applied to the normalized expression data matrix (log scale).

### Differential expression analysis between erlotinib-resistant and -sensitive cell lines

The expression values of biological sample duplicates were averaged and mean expression differences were calculated as positive and negative fold-changes of gene abundance between erlotinib-resistant and -sensitive cell lines. We used -1 and 1 log fold-change cutoffs in order to select genes with relevant down- and up-regulation, respectively. Recurrence in gene expression modulation of erlotinib-resistant cell lines were evaluated by selecting genes with down- or up-regulation in at least five of the six derived vs parental cell lines comparisons. The recurrent differentially expressed genes were ranked according their log fold-change mean in the 5/6 erlotinib-resistant cell lines.

### Selection of EMT-related genes differentially expressed in NSCLC cell lines

Five gene lists previously associated to EMT (Byers et al., 2013 [38]; Gröger et al., 2012 [39]; Huang et al., 2013 [15]; PAHS-090 [40]; R&D System [41]) (Table 4) were pooled together (335 EMT-related genes). Our microarray data set includes 270/335 genes (hereafter the COMBO_270 list) that were further investigated by three different methods: (i.) cut-off on gene expression (CO); (ii.) support vector machine (SVM); (iii.) expression profile analysis (PROF).

Cut-off on gene expression (CO). This method selects genes differentially expressed between epithelial and mesenchymal cell lines (fold-change ≥ 2) and with no modulation between the subset of epithelial cell lines (fold-change within epithelial cell lines lower than 1.5). This method selected a list of 59 genes (hereafter, the CO_59 list).

Support Vector Machines classification (SVM). A supervised classification of cell lines was performed by using the SVM implemented by the Weka software [70]. Based on this classifier, each gene from the COMBO_270 list was assigned a separation hyperplane coefficient for the following three pairwise problems: epithelial vs E/M, E/M vs mesenchymal, and epithelial vs mesenchymal. Genes with all three coefficients greater than 0.01 were selected (hereafter, the SVM_85 list).

Expression profile analysis (PROF). A hierarchical clustering was firstly performed to group genes of the COMBO_270 list according to their expression profiles. The resulting cluster dendrogram was cut at N=38 clusters and expression trends of each cluster separately evaluated. Next, genes populating clusters showing modulation between epithelial and mesenchymal cell lines and/or between E/M phenotype cell lines and both epithelial and mesenchymal cell lines were selected (hereafter the PROF_58 list).

Finally, intersection of the above subsets of selected genes yielded a shared list of 25 genes related to EMT (hereafter INT_25 list).

### Functional enrichment analysis of gene sets

Differentially expressed genes between erlotinib-resistant and -sensitive cell lines and EMT-related gene sets were submitted to the DAVID tool suite [71] to analyze functional enrichment in selected pathways by testing annotation terms provided by different sources, such as KEGG (Kyoto Encyclopedia of Genes and Genomes), PANTHER, BioCarta, Reactome and BBID (Biological Biochemical Image Database). Annotation terms with p-value < 0.05 were considered relevant. As regarding the selected EMT-related gene lists (i.e. CO_59, SVM_85 and PROF_58) gene sets, the annotation relevant terms had p-value lower than 0.05 and fold-enrichment higher than the ones of COMBO_270 gene list.

### Statistical analysis

Statistical analysis was performed using Microsoft Excel and GraphPad Prism software 6.0c. Two-tailed t-test was used for all indicated comparisons if not otherwise stated and p-values lower than 0.05 were considered significant. In particular, the effects of treatment with LY-364947, TGF β1 and their combination were evaluated by analyzing the fold-changes of Vimentin positive cells in vehicle (control) vs LY-364947, vehicle vs TGF β1, and TGF β1 vs LY-364947+ TGF β1 comparisons. In the wound healing assay, differences between leading edge and inner side were evaluated by comparing the counts of total Vimentin positive, Vimentin/Cadherin-1 double positive, and Vimentin positive/Cadherin-1 negative cells. In qPCR experiments resistant cell lines were compared with their corresponding parental cell lines. Statistical analysis of the invasion and sphere-forming assays data was performed using the non-parametric Mann-Whitney U test.

## SUPPLEMENTARY MATERIALS FIGURES AND TABLES









## References

[1] Nieto MA (2013). Epithelial plasticity: a common theme in embryonic and cancer cells. Science.

[2] Thiery JP, Acloque H, Huang RY, Nieto MA (2009). Epithelial-mesenchymal transitions in development and disease. Cell.

[3] Lamouille S, Xu J, Derynck R (2014). Molecular mechanisms of epithelial-mesenchymal transition. Nat Rev Mol Cell Biol.

[4] Christiansen JJ, Rajasekaran AK (2006). Reassessing epithelial to mesenchymal transition as a prerequisite for carcinoma invasion and metastasis. Cancer Res.

[5] Mani SA, Guo W, Liao MJ, Eaton EN, Ayyanan A, Zhou AY, Brooks M, Reinhard F, Zhang CC, Shipitsin M, Campbell LL, Polyak K, Brisken C (2008). The epithelial-mesenchymal transition generates cells with properties of stem cells. Cell.

[6] Nakaya Y, Sheng G (2013). EMT in developmental morphogenesis. Cancer Lett.

[7] Fischer KR, Durrans A, Lee S, Sheng J, Li F, Wong ST, Choi H, El Rayes T, Ryu S, Troeger J, Schwabe RF, Vahdat LT, Altorki NK (2015). Epithelial-to-mesenchymal transition is not required for lung metastasis but contributes to chemoresistance. Nature.

[8] Zheng X, Carstens JL, Kim J, Scheible M, Kaye J, Sugimoto H, Wu CC, LeBleu VS, Kalluri R (2015). Epithelial-to-mesenchymal transition is dispensable for metastasis but induces chemoresistance in pancreatic cancer. Nature.

[9] De Craene B, Berx G (2013). Regulatory networks defining EMT during cancer initiation and progression. Nat Rev Cancer.

[10] Thiery JP, Sleeman JP (2006). Complex networks orchestrate epithelial-mesenchymal transitions. Nat Rev Mol Cell Biol.

[11] Tam WL, Weinberg RA (2013). The epigenetics of epithelial-mesenchymal plasticity in cancer. Nat Med.

[12] Sciacovelli M, Goncalves E, Johnson TI, Zecchini VR, da Costa AS, Gaude E, Drubbel AV, Theobald SJ, Abbo SR, Tran MG, Rajeeve V, Cardaci S, Foster S (2016). Fumarate is an epigenetic modifier that elicits epithelial-to-mesenchymal transition. Nature.

[13] Yu M, Bardia A, Wittner BS, Stott SL, Smas ME, Ting DT, Isakoff SJ, Ciciliano JC, Wells MN, Shah AM, Concannon KF, Donaldson MC, Sequist LV (2013). Circulating breast tumor cells exhibit dynamic changes in epithelial and mesenchymal composition. Science.

[14] Schliekelman MJ, Taguchi A, Zhu J, Dai X, Rodriguez J, Celiktas M, Zhang Q, Chin A, Wong CH, Wang H, McFerrin L, Selamat SA, Yang C (2015). Molecular portraits of epithelial, mesenchymal, and hybrid States in lung adenocarcinoma and their relevance to survival. Cancer Res.

[15] Huang RY, Wong MK, Tan TZ, Kuay KT, Ng AH, Chung VY, Chu YS, Matsumura N, Lai HC, Lee YF, Sim WJ, Chai C, Pietschmann E (2013). An EMT spectrum defines an anoikis-resistant and spheroidogenic intermediate mesenchymal state that is sensitive to e-cadherin restoration by a src-kinase inhibitor, saracatinib (AZD0530). Cell Death Dis.

[16] Sampson VB, David JM, Puig I, Patil PU, de Herreros AG, Thomas GV, Rajasekaran AK (2014). Wilms' tumor protein induces an epithelial-mesenchymal hybrid differentiation state in clear cell renal cell carcinoma. PLoS One.

[17] Revenu C, Gilmour D (2009). EMT 2.0: shaping epithelia through collective migration. Curr Opin Genet Dev.

[18] Jolly MK, Boareto M, Huang B, Jia D, Lu M, Ben-Jacob E, Onuchic JN, Levine H (2015). Implications of the hybrid epithelial/mesenchymal phenotype in metastasis. Front Oncol.

[19] Cheung KJ, Ewald AJ (2016). A collective route to metastasis: Seeding by tumor cell clusters. Science.

[20] Ferlay J, Soerjomataram I, Dikshit R, Eser S, Mathers C, Rebelo M, Parkin DM, Forman D, Bray F (2015). Cancer incidence and mortality worldwide: sources, methods and major patterns in GLOBOCAN 2012. Int J Cancer.

[21] Ding L, Getz G, Wheeler DA, Mardis ER, McLellan MD, Cibulskis K, Sougnez C, Greulich H, Muzny DM, Morgan MB, Fulton L, Fulton RS, Zhang Q (2008). Somatic mutations affect key pathways in lung adenocarcinoma. Nature.

[22] Imielinski M, Berger AH, Hammerman PS, Hernandez B, Pugh TJ, Hodis E, Cho J, Suh J, Capelletti M, Sivachenko A, Sougnez C, Auclair D, Lawrence MS (2012). Mapping the hallmarks of lung adenocarcinoma with massively parallel sequencing. Cell.

[23] Govindan R, Ding L, Griffith M, Subramanian J, Dees ND, Kanchi KL, Maher CA, Fulton R, Fulton L, Wallis J, Chen K, Walker J, McDonald S (2012). Genomic landscape of non-small cell lung cancer in smokers and never-smokers. Cell.

[24] Camidge DR, Pao W, Sequist LV (2014). Acquired resistance to TKIs in solid tumours: learning from lung cancer. Nat Rev Clin Oncol.

[25] Pao W, Chmielecki J (2010). Rational, biologically based treatment of EGFR-mutant non-small-cell lung cancer. Nat Rev Cancer.

[26] Neel DS, Bivona TG (2013). Secrets of drug resistance in NSCLC exposed by new molecular definition of EMT. Clin Cancer Res.

[27] Shien K, Toyooka S, Yamamoto H, Soh J, Jida M, Thu KL, Hashida S, Maki Y, Ichihara E, Asano H, Tsukuda K, Takigawa N, Kiura K (2013). Acquired resistance to EGFR inhibitors is associated with a manifestation of stem cell-like properties in cancer cells. Cancer Res.

[28] Thomson S, Buck E, Petti F, Griffin G, Brown E, Ramnarine N, Iwata KK, Gibson N, Haley JD (2005). Epithelial to mesenchymal transition is a determinant of sensitivity of non-small-cell lung carcinoma cell lines and xenografts to epidermal growth factor receptor inhibition. Cancer Res.

[29] Sequist LV, Waltman BA, Dias-Santagata D, Digumarthy S, Turke AB, Fidias P, Bergethon K, Shaw AT, Gettinger S, Cosper AK, Akhavanfard S, Heist RS, Temel J (2011). Genotypic and histological evolution of lung cancers acquiring resistance to EGFR inhibitors. Sci Transl Med.

[30] Presutti D, Santini S, Cardinali B, Papoff G, Lalli C, Samperna S, Fustaino V, Giannini G, Ruberti G (2015). MET gene amplification and MET receptor activation are not sufficient to predict efficacy of combined MET and EGFR inhibitors in EGFR TKI-resistant NSCLC cells. PLoS One.

[31] Yoshida T, Song L, Bai Y, Kinose F, Li J, Ohaegbulam KC, Munoz-Antonia T, Qu X, Eschrich S, Uramoto H, Tanaka F, Nasarre P, Gemmill RM (2016). ZEB1 mediates acquired resistance to the epidermal growth factor receptor-tyrosine kinase inhibitors in non-small cell lung cancer. PLoS One.

[32] Peinado H, Olmeda D, Cano A (2007). Snail, Zeb and bHLH factors in tumour progression: an alliance against the epithelial phenotype?. Nat Rev Cancer.

[33] Gregory PA, Bert AG, Paterson EL, Barry SC, Tsykin A, Farshid G, Vadas MA, Khew-Goodall Y, Goodall GJ (2008). The miR-200 family and miR-205 regulate epithelial to mesenchymal transition by targeting ZEB1 and SIP1. Nat Cell Biol.

[34] Park SM, Gaur AB, Lengyel E, Peter ME (2008). The miR-200 family determines the epithelial phenotype of cancer cells by targeting the E-cadherin repressors ZEB1 and ZEB2. Genes Dev.

[35] Brabletz S, Brabletz T (2010). The ZEB/miR-200 feedback loop - a motor of cellular plasticity in development and cancer?. EMBO Rep.

[36] Scarpa E, Mayor R (2016). Collective cell migration in development. J Cell Biol.

[37] Theveneau E, Mayor R (2013). Collective cell migration of epithelial and mesenchymal cells. Cell Mol Life Sci.

[38] Byers LA, Diao L, Wang J, Saintigny P, Girard L, Peyton M, Shen L, Fan Y, Giri U, Tumula PK, Nilsson MB, Gudikote J, Tran H (2013). An epithelial-mesenchymal transition gene signature predicts resistance to EGFR and PI3K inhibitors and identifies Axl as a therapeutic target for overcoming EGFR inhibitor resistance. Clin Cancer Res.

[39] Groger CJ, Grubinger M, Waldhor T, Vierlinger K, Mikulits W (2012). Meta-analysis of gene expression signatures defining the epithelial to mesenchymal transition during cancer progression. PLoS One.

[40] Qiagen (2012). https://www.sabiosciences.com/rt_pcr_product/HTML/PAHS-090A.html.

[41] R&D Systems (2014). https://www.rndsystems.com/research-area/epithelial-to-mesenchymal-transition.

[42] Wong CH, Wu Z, Yu Q (2014). CTSL2 is a pro-apoptotic target of E2F1 and a modulator of histone deacetylase inhibitor and DNA damage-induced apoptosis. Oncogene.

[43] Santamaria I, Velasco G, Cazorla M, Fueyo A, Campo E, Lopez-Otin C (1998). Cathepsin L2, a novel human cysteine proteinase produced by breast and colorectal carcinomas. Cancer Res.

[44] Ameshima S, Ishizaki T, Demura Y, Imamura Y, Miyamori I, Mitsuhashi H (2000). Increased secretory leukoprotease inhibitor in patients with nonsmall cell lung carcinoma. Cancer.

[45] Barker SD, Coolidge CJ, Kanerva A, Hakkarainen T, Yamamoto M, Liu B, Rivera AA, Bhoola SM, Barnes MN, Alvarez RD, Curiel DT, Hemminki A (2003). The secretory leukoprotease inhibitor (SLPI) promoter for ovarian cancer gene therapy. J Gene Med.

[46] Israeli O, Goldring-Aviram A, Rienstein S, Ben-Baruch G, Korach J, Goldman B, Friedman E (2005). In silico chromosomal clustering of genes displaying altered expression patterns in ovarian cancer. Cancer Genet Cytogenet.

[47] Koshikawa N, Nakamura T, Tsuchiya N, Isaji M, Yasumitsu H, Umeda M, Miyazaki K (1996). Purification and identification of a novel and four known serine proteinase inhibitors secreted by human glioblastoma cells. J Biochem.

[48] Rasool N, LaRochelle W, Zhong H, Ara G, Cohen J, Kohn EC (2010). Secretory leukocyte protease inhibitor antagonizes paclitaxel in ovarian cancer cells. Clin Cancer Res.

[49] Yang D, Holt GE, Velders MP, Kwon ED, Kast WM (2001). Murine six-transmembrane epithelial antigen of the prostate, prostate stem cell antigen, and prostate-specific membrane antigen: prostate-specific cell-surface antigens highly expressed in prostate cancer of transgenic adenocarcinoma mouse prostate mice. Cancer Res.

[50] Hubert RS, Vivanco I, Chen E, Rastegar S, Leong K, Mitchell SC, Madraswala R, Zhou Y, Kuo J, Raitano AB, Jakobovits A, Saffran DC, Afar DE (1999). STEAP: a prostate-specific cell-surface antigen highly expressed in human prostate tumors. Proc Natl Acad Sci U S A.

[51] Gomes IM, Maia CJ, Santos CR (2012). STEAP proteins: from structure to applications in cancer therapy. Mol Cancer Res.

[52] Zhuang X, Herbert JM, Lodhia P, Bradford J, Turner AM, Newby PM, Thickett D, Naidu U, Blakey D, Barry S, Cross DA, Bicknell R (2015). Identification of novel vascular targets in lung cancer. Br J Cancer.

[53] Kasai H, Allen JT, Mason RM, Kamimura T, Zhang Z (2005). TGF-beta1 induces human alveolar epithelial to mesenchymal cell transition (EMT). Respir Res.

[54] Wilson C, Nicholes K, Bustos D, Lin E, Song Q, Stephan JP, Kirkpatrick DS, Settleman J (2014). Overcoming EMT-associated resistance to anti-cancer drugs via Src/FAK pathway inhibition. Oncotarget.

[55] Yao Z, Fenoglio S, Gao DC, Camiolo M, Stiles B, Lindsted T, Schlederer M, Johns C, Altorki N, Mittal V, Kenner L, Sordella R (2010). TGF-beta IL-6 axis mediates selective and adaptive mechanisms of resistance to molecular targeted therapy in lung cancer. Proc Natl Acad Sci U S A.

[56] Ciardiello C, Roca MS, Noto A, Bruzzese F, Moccia T, Vitagliano C, Di Gennaro E, Ciliberto G, Roscilli G, Aurisicchio L, Marra E, Mancini R, Budillon A (2016). Synergistic antitumor activity of histone deacetylase inhibitors and anti-ErbB3 antibody in NSCLC primary cultures via modulation of ErbB receptors expression. Oncotarget.

[57] Roca H, Hernandez J, Weidner S, McEachin RC, Fuller D, Sud S, Schumann T, Wilkinson JE, Zaslavsky A, Li H, Maher CA, Daignault-Newton S, Healy PN (2013). Transcription factors OVOL1 and OVOL2 induce the mesenchymal to epithelial transition in human cancer. PLoS One.

[58] Jia D, Jolly MK, Boareto M, Parsana P, Mooney SM, Pienta KJ, Levine H, Ben-Jacob E (2015). OVOL guides the epithelial-hybrid-mesenchymal transition. Oncotarget.

[59] Hong T, Watanabe K, Ta CH, Villarreal-Ponce A, Nie Q, Dai X (2015). An Ovol2-Zeb1 Mutual inhibitory circuit governs bidirectional and multi-step transition between epithelial and mesenchymal states. PLoS Comput Biol.

[60] Chung VY, Tan TZ, Tan M, Wong MK, Kuay KT, Yang Z, Ye J, Muller J, Koh CM, Guccione E (2016). GRHL2-miR-200-ZEB1 maintains the epithelial status of ovarian cancer through transcriptional regulation and histone modification. Sci Rep.

[61] Mayor R, Etienne-Manneville S (2016). The front and rear of collective cell migration. Nat Rev Mol Cell Biol.

[62] Mosmann T (1983). Rapid colorimetric assay for cellular growth and survival: application to proliferation and cytotoxicity assays. J Immunol Methods.

[63] Hansen MB, Nielsen SE, Berg K (1989). Re-examination and further development of a precise and rapid dye method for measuring cell growth/cell kill. J Immunol Methods.

[64] Schindelin J, Rueden CT, Hiner MC, Eliceiri KW (2015). The ImageJ ecosystem: An open platform for biomedical image analysis. Mol Reprod Dev.

[65] Murakami A, Takahashi F, Nurwidya F, Kobayashi I, Minakata K, Hashimoto M, Nara T, Kato M, Tajima K, Shimada N, Iwakami S, Moriyama M, Moriyama H (2014). Hypoxia increases gefitinib-resistant lung cancer stem cells through the activation of insulin-like growth factor 1 receptor. PLoS One.

[66] Chou TC (2010). Drug combination studies and their synergy quantification using the Chou-Talalay method. Cancer Res.

[67] Chou T, Martin N (2005). CompuSyn for drug combinations: PC software and user’s guide: a computer program for quantitation of synergism and antagonism in drug combinations, and the determination of IC50 and ED50 and LD50 values.

[68] Ritchie ME, Phipson B, Wu D, Hu Y, Law CW, Shi W, Smyth GK (2015). limma powers differential expression analyses for RNA-sequencing and microarray studies. Nucleic Acids Res.

[69] Edgar R, Domrachev M, Lash AE (2002). Gene Expression Omnibus: NCBI gene expression and hybridization array data repository. Nucleic Acids Res.

[70] Hall M, Frank E, Holmes G, Pfahringer B, Reutemann P, Witten IH (2009). The WEKA data mining software: an update. ACM SIGKDD explorations newsletter.

[71] Huang DW, Sherman BT, Lempicki RA (2009). Systematic and integrative analysis of large gene lists using DAVID bioinformatics resources. Nat Protoc.

