# Supplementary material for: Characterization of epithelial-mesenchymal transition intermediate/hybrid phenotypes associated to resistance to EGFR inhibitors in non-small cell lung cancer cell lines
Source: Oncotarget. 2017 Sep 22;8(61):103340–63. doi: 10.18632/oncotarget.21132 (PMC5732732; doi:10.18632/oncotarget.21132)
Supplement: Supplementary file 1 [file oncotarget-08-103340-s001.pdf]

Characterization of epithelial-mesenchymal transition intermediate/hybrid phenotypes associated to resistance to EGFR inhibitors in non-small cell lung cancer cell lines

SUPPLEMENTARY MATERIALS

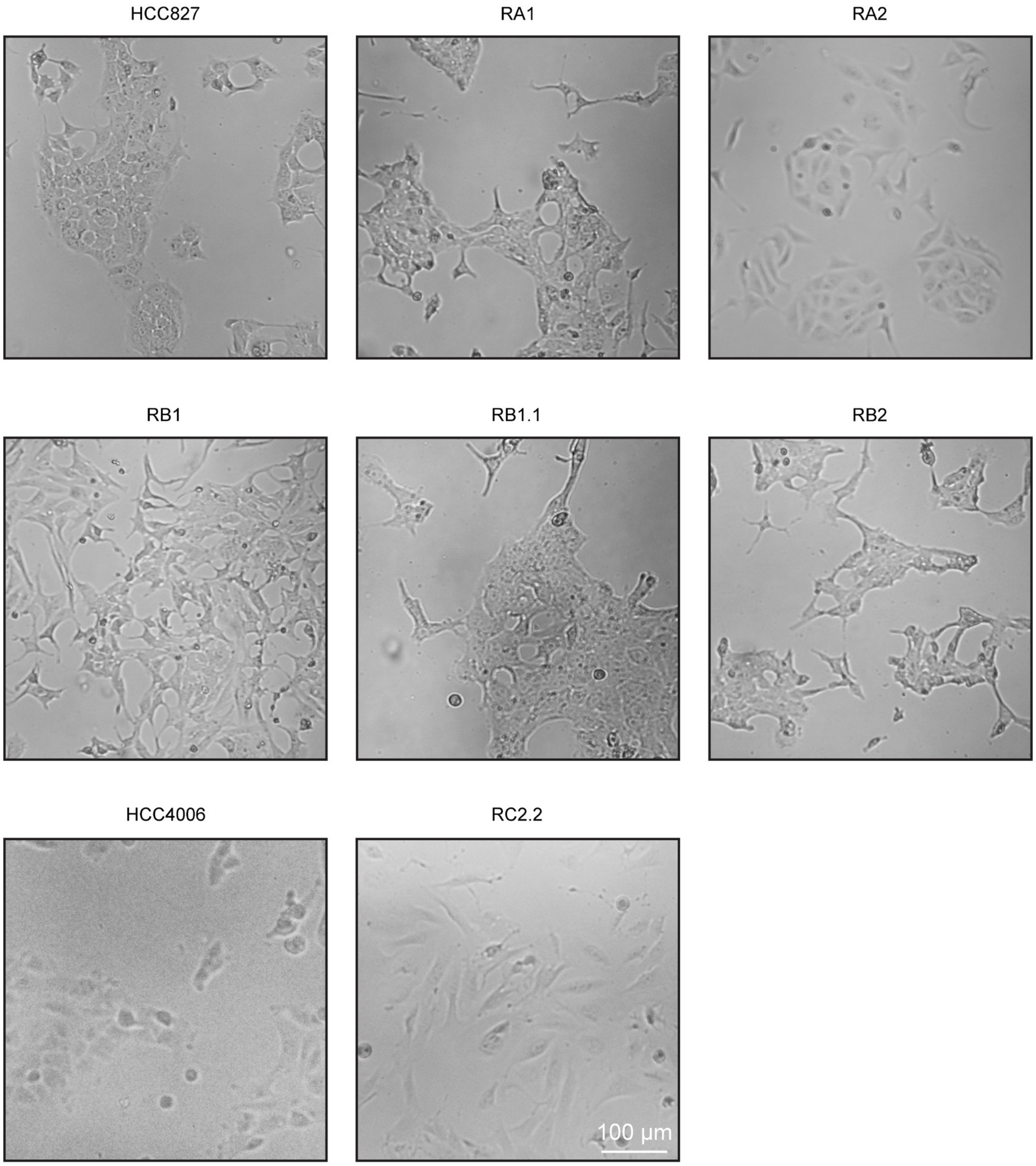

Supplementary Figure 1: Morphology of NSCLC cell lines. Erlotinib-sensitive and -resistant cell lines were cultured at low confluence and analyzed with a Leica DM1L microscope with a 4x objective. Scale bar is indicated.

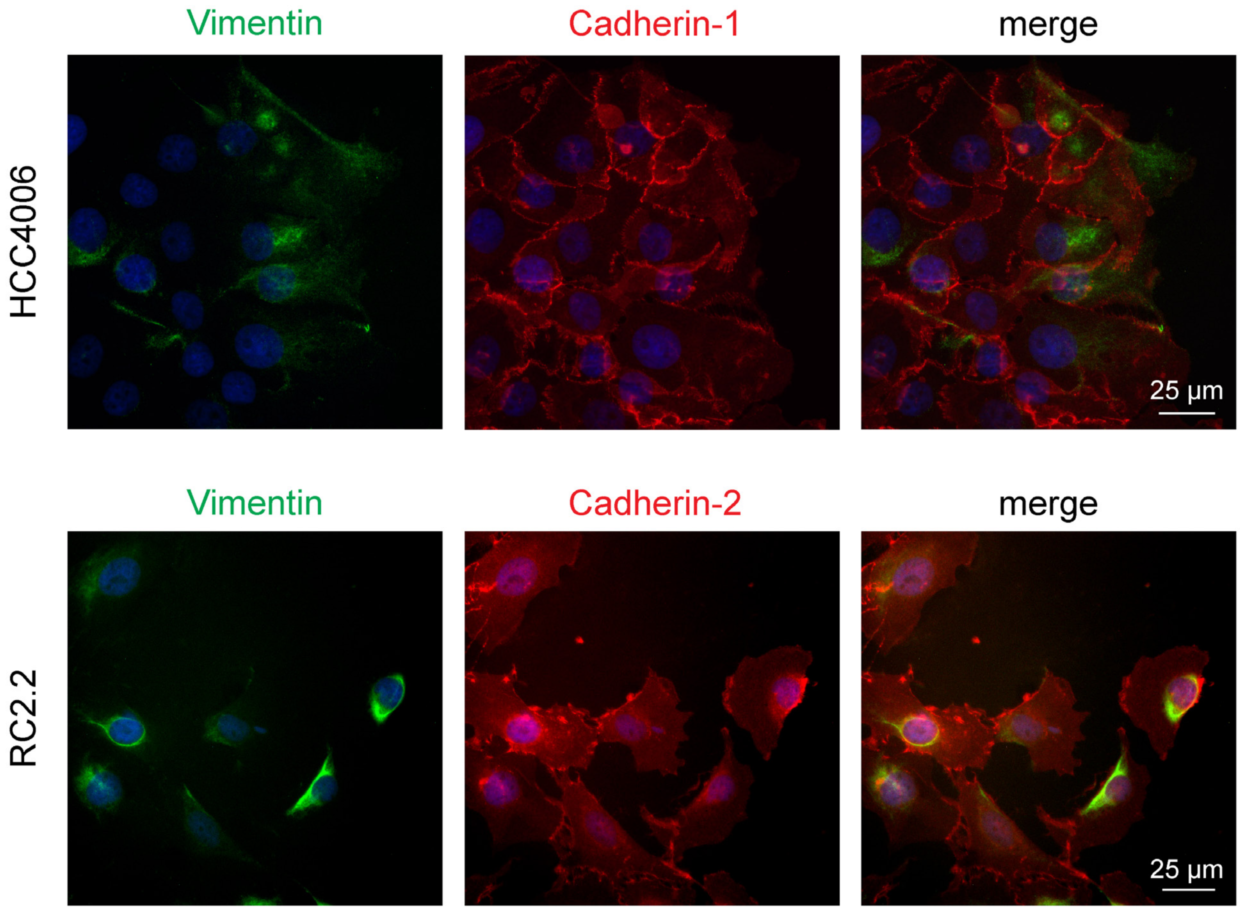

Supplementary Figure 2: Cell migration of HCC4006 and RC2.2. cell lines at the wound leading edge. Representative fluorescence microscopy images of wound healing of HCC4006 (erlotinib-sensitive) and RC2.2 (erlotinib-resistant) cell lines stained with specific reactive antibodies for Vimentin (green), Cadherin-1 (HCC4006) or Cadherin-2 (RC2.2) (red) and Hoechst 33258 for nuclei (blue). Scale bars are indicated.

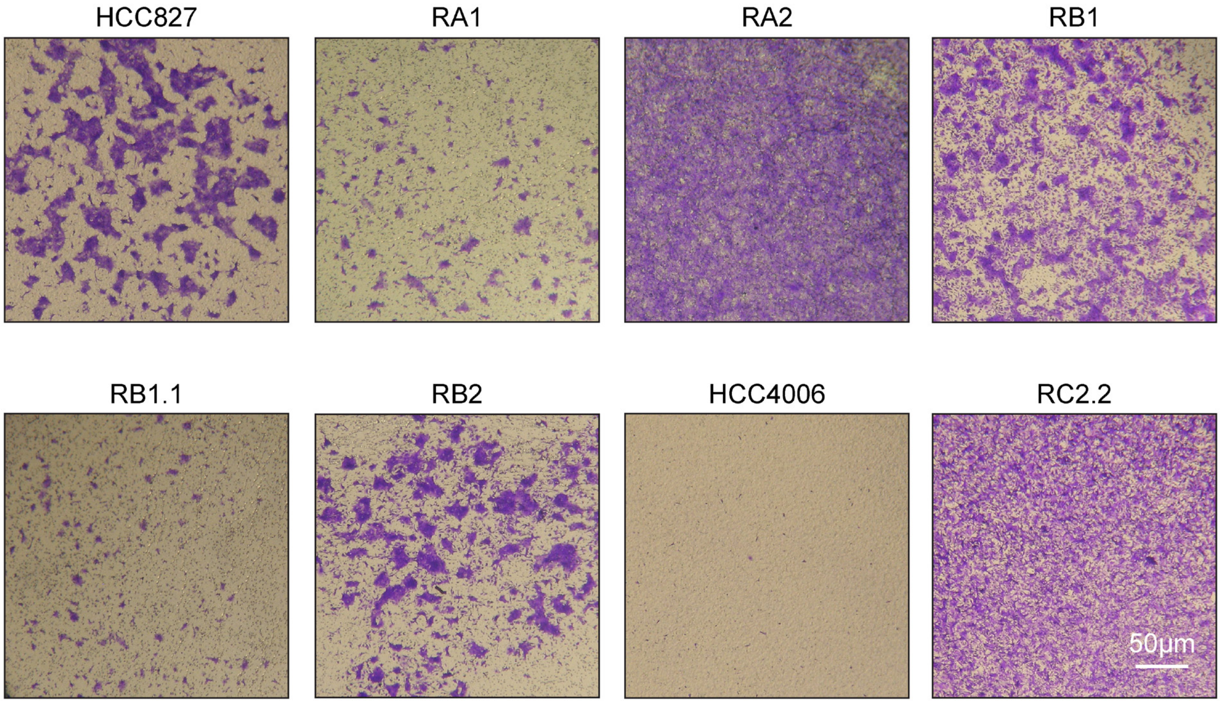

Supplementary Figure 3: Cell invasion of erlotinib-sensitive and -resistant NSCLC cell lines. Light microscopy images. Representative fields of erlotinib-sensitive (HCC827 and HCC4006) and –resistant (HCC827-derived RA1, RA2, RB1, RB1.1, RB2 and HCC4006-derived RC2.2) invasive cells on the membrane filter stained with crystal violet are shown. Scale bar is indicated.

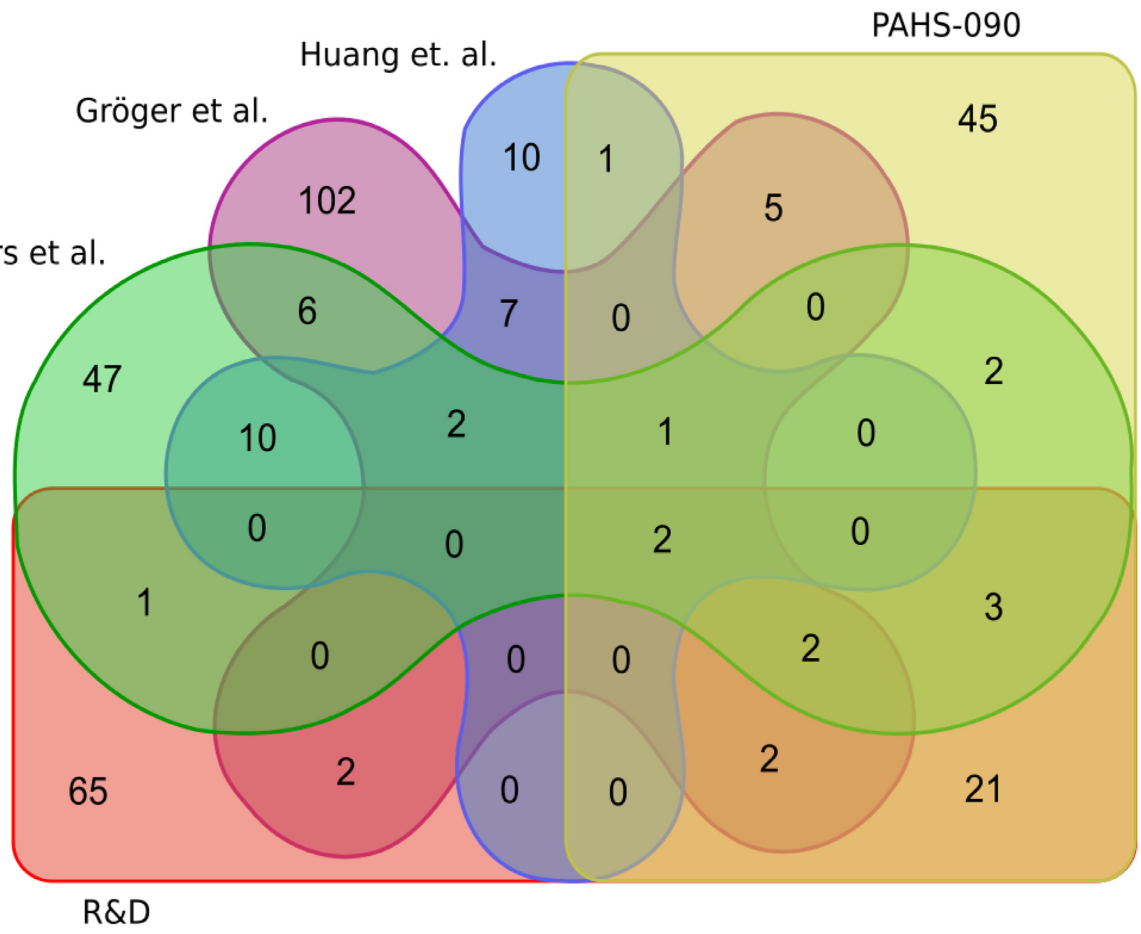

Supplementary Figure 4: Venn diagram of the five EMT-related gene lists. The union of all gene lists (as described in Table 4) was 335 genes.

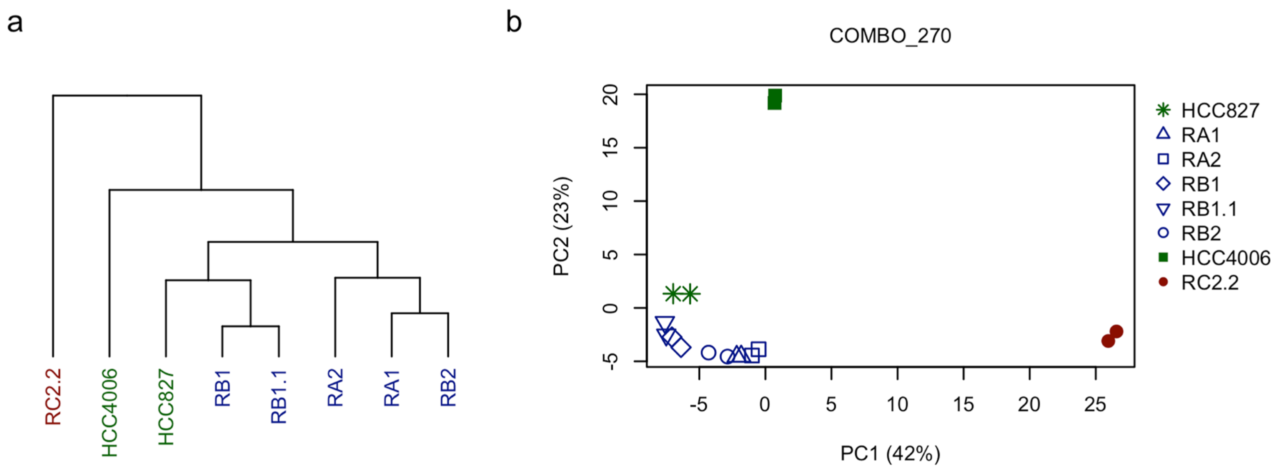

Supplementary Figure 5: Analysis of the expression of EMT-related genes in erlotinib-resistant NSCLC cell lines. Hierarchical clustering dendrogram **(a)** and PC plot **(b)** of the NSCLC cell lines based on the expression of the COMBO_270 EMT-related genes. In (b), the percentage of the PC explained variance is shown in brackets. The colors indicate the EMT phenotype features, in green epithelial, in blue epithelial/mesenchymal and in red mesenchymal.

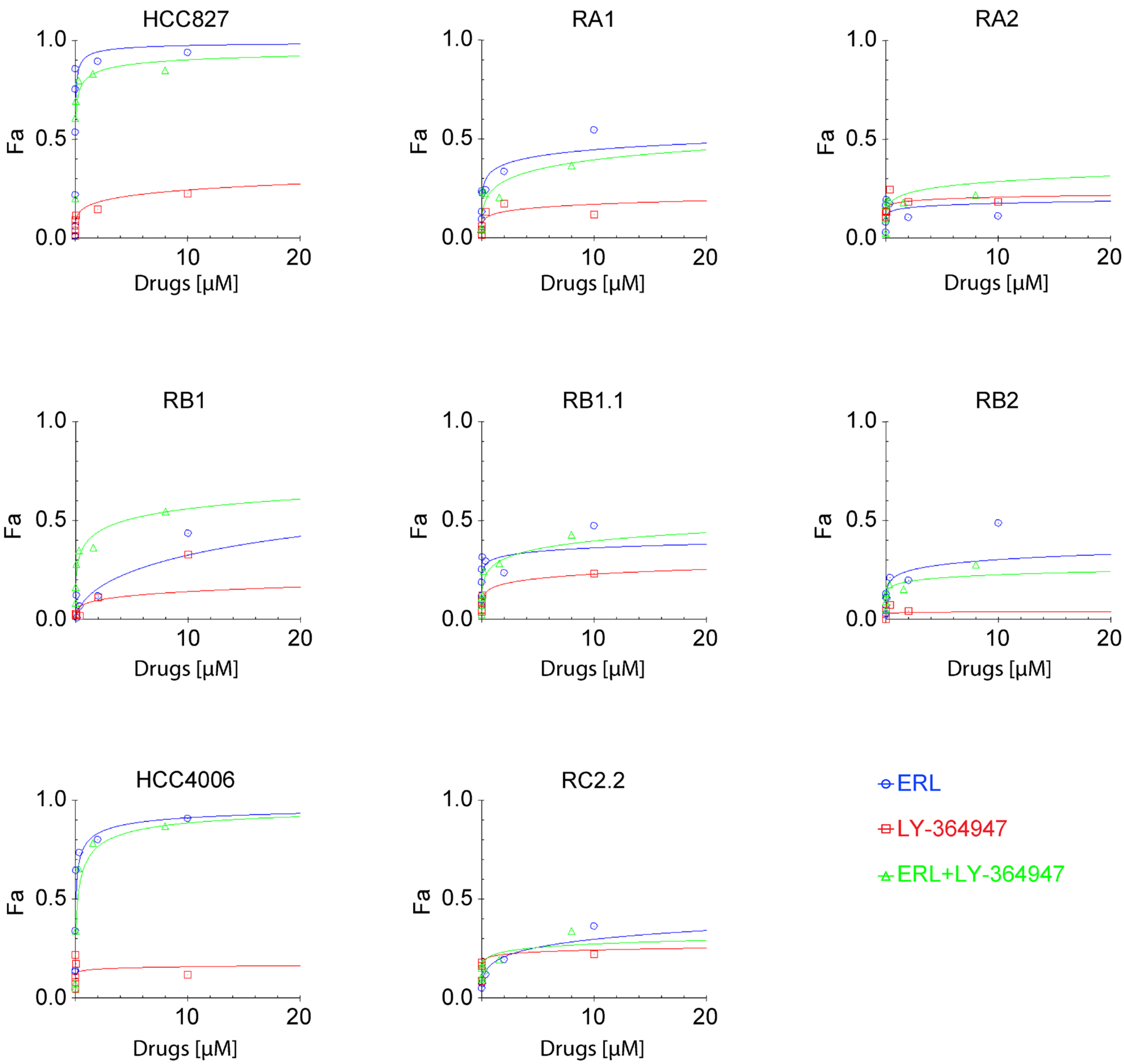

Supplementary Figure 6: Effects of erlotinib and TGFRβ-1 inhibitors on NSCLC cell lines survival. Dose-effect curves were calculated according to the Chou-Talalay method as described in material and methods. The curves show the fraction of cells affected by the treatment (Fa) in function of drug concentration. For the double-drug treatments, the combined drug concentrations were used. Each data point represents the mean of 3 replicates.

Supplementary Table 1: Features of HCC827 and HCC4006 erlotinib-resistant derived cell lines

	HCC827	RA1	RA2	RB1	RB1.1	RB2	HCC4006	RC2.2
EGFR mutations in Exon 19	Y	Y	Y	Y	Y	Y	Y	Y
EGFR gene amplification	Y	Y	N	Y	Y	Y	Y	Y
EGFR T790M mutation	N	N	N	N	N	N	N	N
Soft Agar growth	Y	Y	Y	Y	Y	Y	Y	Y
Xenograft growth	Y	Y	Y	Y	Y	Y	Y	Y
HER2 gene amplification	N	N	N	N	N	N	N	N
HER3 gene amplification	N	N	N	N	N	N	N	N
MET gene amplification	N	Y	N	Y	Y	Y	N	N
AXL upregulation	N	Y	Y	Y	Y	Y	N	Y
EMT morphology	N	Y	Y	Y	Y	Y	N	Y
pEGFR downregulation upon ERL treatment	Y	Y	Y	Y	Y	Y	Y	Y
pERK1/2 downregulation upon ERL treatment	Y	Y	Y	Y	Y	Y	Y	Y
Effect of ERL+ AXL inhibitors on cell survival	N	N	N	N	N	N	N	N
Effect of ERL+MET TK inhibitors on cell survival	N	N	N	N	Y	N	N	N

Y = yes and N = no; pEGFR and pERK1/2 indicate respectively phosphorylation in tyrosine 1068 and threonine 202/ tyrosine 204; ERL indicates erlotinib, TK tyrosine kinase

Supplementary Table 2: Expression values (log scale), log fold-changes and annotations of genes differentially expressed in erlotinib-resistant NSCLC cell lines

See Supplementary File 1

Supplementary Table 3: Pathway annotations of the INT_25 gene list

See Supplementary File 2

Supplementary Table 4: Expression values (log scale) and log fold-changes of genes coding for stemness markers

See Supplementary File 3
